# Synergistic Interactions among *Burkholderia cepacia* Complex-Targeting Phages Reveal a Novel Therapeutic Role for Lysogenization-Capable Phages

**DOI:** 10.1128/spectrum.04430-22

**Published:** 2023-05-17

**Authors:** Philip Lauman, Jonathan J. Dennis

**Affiliations:** a Department of Biological Sciences, University of Alberta, Edmonton, Alberta, Canada; University of Pittsburgh School of Medicine

**Keywords:** bacteriophage, phage therapy, lysogeny, lysogenization, *Burkholderia cepacia* complex, bacteriophage therapy

## Abstract

Antimicrobial resistance is a danger to global public health and threatens many aspects of modern medicine. Bacterial species such as those of the Burkholderia cepacia complex (Bcc) cause life-threatening respiratory infections and are highly resistant to antibiotics. One promising alternative being explored to combat Bcc infections is phage therapy (PT): the use of phages to treat bacterial infections. Unfortunately, the utility of PT against many pathogenic species is limited by its prevailing paradigm: that only obligately lytic phages should be used therapeutically. It is thought that ‘lysogenic’ phages do not lyse all bacteria and can transfer antimicrobial resistance or virulence factors to their hosts. We argue that the tendency of a lysogenization-capable (LC) phage to form stable lysogens is not predicated exclusively on its ability to do so, and that the therapeutic suitability of a phage must be evaluated on a case-by-case basis. Concordantly, we developed several novel metrics—Efficiency of Phage Activity, Growth Reduction Coefficient, and Stable Lysogenization Frequency—and used them to evaluate eight Bcc-specific phages. Although these parameters vary considerably among Bcc phages, a strong inverse correlation (R^2^ = 0.67; *P* < 0.0001) exists between lysogen formation and antibacterial activity, indicating that certain LC phages with low frequency of stable lysogenization may be therapeutically efficacious. Moreover, we show that many LC Bcc phages interact synergistically with other phages in the first reported instance of mathematically defined polyphage synergy, and that these interactions result in the eradication of *in vitro* bacterial growth. Together, these findings reveal a novel therapeutic role for LC phages and challenge the current paradigm of PT.

**IMPORTANCE** The spread of antimicrobial resistance is an imminent threat to public health around the world. Particularly concerning are species of the Burkholderia cepacia complex (Bcc), which cause life-threatening respiratory infections and are notoriously resistant to antibiotics. Phage therapy is a promising alternative being explored to combat Bcc infections and antimicrobial resistance in general, but its utility against many pathogenic species, including the Bcc, is restricted by the currently prevailing paradigm of exclusively using rare obligately lytic phages due to the perception that ‘lysogenic’ phages are therapeutically unsuitable. Our findings show that many lysogenization-capable phages exhibit powerful *in vitro* antibacterial activity both alone and through mathematically defined synergistic interactions with other phages, demonstrating a novel therapeutic role for LC phages and therefore challenging the currently prevailing paradigm of PT.

## INTRODUCTION

The increasing prevalence and global distribution of multidrug-resistant (MDR) bacterial pathogens is an imminent menace to public health and threatens virtually all aspects of modern medicine. Antibiotic resistance is a major cause of mortality in both developed and developing countries worldwide, and it is currently predicted that drug resistance will cause over 10 million premature deaths per year by 2050 and cost approximately 100 USD trillion in damages (1–3). Discovery of new antibiotics with novel cellular targets has failed to keep pace with the evolution of bacterial resistance, which has now emerged even against last-resort antibiotics, meaning that novel modes of treatment are desperately needed to combat resistant pathogens (4, 5).

Among the most problematic of these pathogens are the members of the Burkholderia cepacia complex (Bcc), a group of over 20 notoriously drug-resistant species which cause severe disease in patients who are immunocompromised or afflicted with certain diseases, notably chronic granulomatous disease (CGD) and cystic fibrosis (CF) (6–8). Although Bcc members account for a relatively small proportion of pulmonary infections in both CF and CGD populations, infections by these organisms are remarkably aggressive and have the highest mortality rates in both of these patient groups. In many cases, these high fatality rates are due to a severe disease progression termed “*cepacia* syndrome,” which contrasts with the clinical progressions of infections caused by other CF and CGD pathogens and is characterized by high fever, severe progressive respiratory failure, declines in leukocyte and erythrocyte levels, necrosis, bacteremia, and sepsis, which result in rapid death (8–12). Moreover, at least five Bcc species, B. cepacia, B. cenocepacia, B. multivorans, B. dolosa, and B. contaminans, can spread via aerosol droplets, meaning that they can disseminate readily, through direct and indirect contact, among susceptible patients. Finally, Bcc species possess a large repertoire of both innate and acquired antimicrobial resistance mechanisms which confer resistance to most antibiotics (7, 8). Even the most effective anti-Bcc antibiotics have been reported to inhibit only 23%s to 38% of clinical isolates in the United States, meaning that conventional antibiotic treatments are largely ineffective, and alternative approaches to treating Bcc infections are therefore urgently required (12–14).

One alternative strategy being explored to combat Bcc infections, and antimicrobial resistance in general, is phage therapy, the clinical administration of bacteriophages (or phages) to treat bacterial infections. Phages are bacterial viruses which readily infect and destroy bacterial cells in order to replicate themselves and are environmentally abundant, outnumbering the bacterial population by approximately 10-fold. Importantly, phage predation produces an estimated 10^23^ infections per second and destroys roughly 50% of the global bacterial population every 48 h, meaning that phage therapy is merely the application of a naturally occurring antibacterial agent to a human problem (8, 15–17). In nature, the tailed phages of the order *Caudovirales*, which are the most relevant to phage therapy, most commonly employ two evolutionarily complementary replication cycles, termed the lytic and lysogenic cycles. In the lytic cycle, phages begin the sequential process of genome replication, protein expression, virion assembly, and cell lysis immediately upon entry into a host cell, resulting in relatively rapid destruction of the cell and, by extension, the host population. In the complementary lysogenic cycle, the phage genome is incorporated into that of the host and replicates passively with the host cell until conditions trigger induction of the lytic cycle. Lysis of the host cell is therefore the ultimate result of both these replication cycles but is generally achieved more slowly through the lysogenic cycle (8, 18, 19).

In keeping with the terminology used to describe the replication cycles of therapeutically relevant *Caudovirales*, these phages have canonically been dichotomously categorized as either “lytic” or “lysogenic,” designations with which the terms “virulent” and “temperate,” respectively, have often been used interchangeably. The current use of this terminology is quite problematic, however, because so-called “lysogenic” phages can still produce lysis and can be virulent in the sense that they kill bacteria or reduce bacterial growth. In recent years, some researchers have begun utilizing the term “obligately lytic” (OL; also called strictly or professionally lytic) to more accurately describe phages that are genetically incapable of forming lysogens, while the term “virulent” has been mathematically defined by Storms et al. as describing phages that are highly capable of reducing bacterial growth during the logarithmic phase (20–22). Nevertheless, similar elaboration has not been achieved thus far for the terms “lysogenic” or “temperate.” Such phages are canonically deemed “lysogenic” on the genetic basis of possessing a lysogenic cassette—a set of genes encoding proteins such as lytic repressors, integrases, and partition systems—or on the experimental basis of being known to produce lysogens on at least one bacterial host strain (12, 23, 24). Because the tendency to form lysogens is not predicated solely on the ability to do so and also depends on host and environmental factors (23, 25), it is inaccurate to say that all phages which are genetically capable of forming lysogens or do so under a single set of environmental conditions also form lysogens at a meaningful frequency under *all* possible environmental conditions, including conditions which are therapeutically relevant. Concordantly, we propose that these phages should be described as lysogenization-capable (LC) rather than lysogenic, while the latter term should be reserved for description of the lysogenic replication cycle. Rather than being dichotomously categorized as either “lytic” or “lysogenic,” individual phages should be understood as occupying points on a spectrum in terms of their tendency to form lysogens under a particular set of environmental conditions, with OL phages occupying one of the outer boundaries of this spectrum. Also, the term “temperate” implicitly suggests a lack of lytic activity and virulence, which is not always the case among lysogenization-capable phages (26–29), and this term should therefore be used as an antonym to “virulent” rather than as a synonym for lysogenization-capable.

Although the antibacterial effects of such LC phages have been explored, both *in vitro* and *in vivo*, in a few studies over the past decade (21, 27, 29–32), the prevailing paradigm within phage therapy is that only OL phages are therapeutically suitable, while LC phages are therapeutically suboptimal. This preference is due to the perception that if they form lysogens, LC phages (i) may not effectively eliminate the targeted bacterial population, (ii) may engage in specialized transduction upon induction to the lytic cycle, and (iii) may provide virulence or antimicrobial resistance factors to target bacteria (8, 20, 21, 23, 33). As previously discussed, however, the mere ability of a phage to produce lysogens is not the sole predictor of whether the phage will do so under therapeutically relevant conditions. Furthermore, even if such phages do form lysogens, they may not have virulence or antimicrobial resistance factors to transfer. Finally, even if certain phages form lysogens under therapeutically relevant conditions, this only implies that they cannot be used on their own through monophage therapy. Combining these phages with OL phages or LC phages which do not form lysogens under therapeutic conditions could still form therapeutically efficacious polyphage cocktails. As a result, LC phages should not be dismissed outright based on their genetic properties or even their ability to form lysogens in a single host, but instead ought to be evaluated for their tendency to form stable lysogens and ability to reduce bacterial growth, both alone and in combination with other phages, on a case-by-case basis.

The aforementioned shortcomings in the current paradigm of phage therapy would be harmless if OL phages were abundant, but these phages are in fact challenging to find for many therapeutically problematic bacterial species, while LC phages appear to be ubiquitous (21, 23, 34). Among the Bcc, for instance, only five OL phages have ever been identified, and only one of these has been characterized (8, 35, 36). This is unsurprising from an evolutionary perspective, since the possession of a lysogenic cassette is evolutionarily beneficial for the long-term stability of phage populations (17, 23, 37, 38). It is reasonable, then, to expect that OL phages may be the minority of all existing phages, and the current paradigm of only using these phages is therefore not only illogical, but also impractical, as it may restrict the range of bacterial species against which phage therapy can be utilized.

It is thus clear that a case-by-case evaluation of individual LC phages is necessary to validate their therapeutic suitability. Furthermore, therapeutic applications of phages are unlikely to consist of a single phage, LC or OL, due to the high risk of the development of resistance (8, 39), and a case-by-case evaluation of how individual LC phages interact with other phages is therefore equally indispensable for evaluating the therapeutic utility of these phages. To further these goals for phages targeting the Bcc—a group of pathogenic species against which polyphage cocktail development is desperately required but has never previously been described in literature—we developed novel metrics to gauge the tendency of Bcc phages to form stable lysogens as well their capacity to reduce bacterial growth *in vitro*, both alone and in combination with another phage. We then investigated these properties for eight previously characterized Bcc phages, one of which is OL and seven of which are known, experimentally and genetically, to be LC, against 17 variably susceptible strains of the Bcc. Here, we present the trends identified for these variables, as well as the relationships between them, and demonstrate that at least some LC phages have enormous therapeutic potential—not only via monophage treatment, but also in combinations in which they synergize with other phages to effectively abolish bacterial growth.

## RESULTS

### Efficiency of Phage Activity is a novel quantitative approach for screening phages on solid medium.

Efficiency of Plaquing (EOP; also called bacteriophage Efficiency of Plating) is a widely used metric for determining a phage’s host range and the efficiency with which it can infect a given host bacterium on solid medium (40–42). While obviously useful, the fact that this approach requires the counting of discrete plaques means that it cannot be used when working with phages which do not form plaques and instead produce turbid clearing or mottling, which is sometimes indicative of nonproductive activity such as abortive infection or lysis from without but can also indicate low levels of productive infection (43, 44). Because many Bcc phages produce such non-plaque evidence of phage activity on certain hosts and therefore cannot be evaluated using the EOP, we devised an alternative approach, designated Efficiency of Phage Activity (EPA), which uses all possible evidence of infection (including mottling, turbid clearing, and plaquing) rather than relying exclusively on plaques. In this approach, the EPA of a phage *x* on a given host strain *h* is computed as the logarithm of the quotient of the lowest order-of-magnitude concentration at which evidence of infection is *theoretically* possible, [*P_x_*]_L(T)_, and the lowest order-of-magnitude concentration at which evidence of infection is *actually* seen, [*P_x_*]_L(A)_, as shown in equation 1:

(1) $${\mathrm{EPA}}_{x(h)}=\log\left(\frac{{\left[P_{x}\right]}_{\mathrm{L(T)}}}{{\left[P_{x}\right]}_{\mathrm{L(A)}}}\right)$$

EPA thus provides an order-of-magnitude estimate of a given phage’s activity on a particular bacterial strain on solid medium, even if it is unable to form discrete plaques. This allows direct comparison of the activities of different phages against particular strains and thus serves as a starting point for evaluating which phage-host pairs can be explored for therapeutic applications. It is crucial to note that while useful, this approach cannot distinguish between phages and antibiotics or bacteriocins, meaning that it should never be used to screen raw bacterial lysates but should only be used with purified stocks containing a single phage type. Using this modified approach, we quantified the EPA of our eight Bcc phages on a panel of 17 strains belonging to five species of the Bcc (Fig. S1 in the supplemental material). We found that the EPA varied substantially by host strain for most tested Bcc phages except for the OL phage JG068, which had a high EPA on all tested host strains. Interestingly, many phages had host ranges which differed from those reported in previous literature (8), suggesting modified host affinities possibly resulting from adaptation in the lab. Since this approach is intended to serve as a preliminary screen to identify phages with high levels of activity against particular strains, we utilized an arbitrary EPA threshold of EPA ≥ −5 and pursued subsequent experiments with phage-host pairs which satisfied this requirement, while those that did not were excluded from further studies.

### The Growth Reduction Coefficient, a novel metric for quantification of a phage’s ability to reduce bacterial growth, reveals enormous diversity in the antibacterial capabilities of Bcc phages.

Because *in vivo* infection dynamics are likely better represented by infections in liquid medium rather than solid medium (22, 42, 45, 46), we sought to evaluate the *in vitro* antibacterial abilities of Bcc phages through planktonic killing assays (PKA). Within the field of phage therapy, quantitative assessments of antibacterial effects using endpoint values are common, but such approaches can be misleading because they provide no data about what occurs during the infection and may therefore under- or overestimate the true antibacterial effect throughout the infection process (22). In addition, studies in which qualitative assessments are made regarding the effects of phage administration on entire bacterial growth curves are abundant (30, 47, 48), but a rigorous mathematical approach to quantitatively interpret these effects was previously unavailable. In a recent study, Storms et al. (22) made a major contribution to this field by developing a novel variable termed the Virulence Index (VI), which quantifies the ability of a phage to reduce bacterial growth during the logarithmic growth phase. Here, we present a therapeutically relevant variant of this metric, designated the Growth Reduction Coefficient (GRC), which quantifies the ability of a phage to reduce bacterial growth across the entirety of a therapeutically relevant period of time, which may vary depending on the typical clinical course of infection by the particular bacterial species being investigated. Because GRC is measured across such an extensive time period, rather than the logarithmic growth phase of the bacterium, it can detect whether a phage can reduce bacterial growth after the stationary phase has been reached or prevent resistant outgrowths which may appear at a later time, an advantage which is particularly relevant when investigating the antibacterial potential of phage-phage or phage-antibiotic combinations. In presenting the VI, Storms et al. found that virulence varies substantially with the growth medium and temperature being utilized (22), so it is logical to assume that similar variability would be found when using the GRC metric. In this study, however, we focused on a particular set of infection conditions which were used in previous *in vitro* work with Bcc phages.

**FIG 1:**
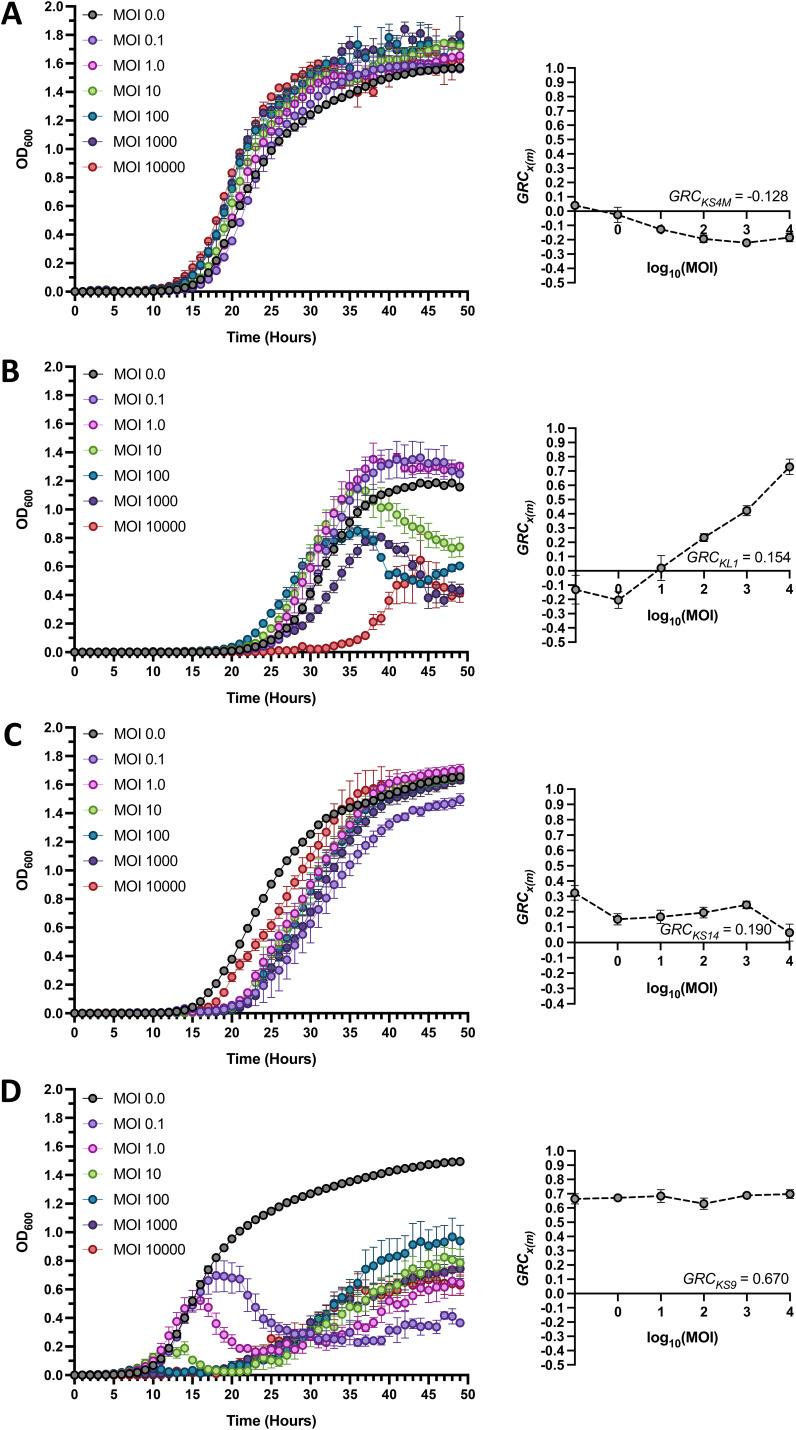
Representative Growth Reduction Trends of Phages Targeting the Bcc. Bacterial growth curves (left) and associated Growth Reduction Coefficient (GRC) curves (right) of various Burkholderia cepacia complex Bcc phage-host pairs for infections conducted under standard conditions across the standard multiplicity of infection (MOI) range of 0.1 to 10,000. Phage-host pairs shown are representative of situations in which the established GRC threshold GRC*_x_* or ${\mathrm{GRC}}_{x_{\max}}$ ≥ 0.05 is not met (panel A: KS4M on B. cenocepacia C6433), and of MOI-GRC trends in which the ${\mathrm{GRC}}_{x_{\max}}$ increases with MOI (panel B: KL1 on B. cenocepacia AU41264), decreases with MOI (panel C: KS14 on B. multivorans C5393), or appears to be relatively independent of the MOI (panel D: KS9 on B. cenocepacia AU41386). Black lines represent bacterial growth without phage (MOI = 0.0), while colored lines represent each of the investigated MOIs. ${\mathrm{GRC}}_{x_{\max}}$ values were computed for each MOI using equation 3; GRC*_x_* values for all representative phage-host pairs were calculated using equation 6 and are shown here for each representative pair. Bars represent standard error of the mean (SEM) of at least three biological replicates.

In this approach, a target bacterial strain is grown in the presence and absence of a phage *x* at various multiplicities of infection (MOI, *m*), and the definite integral of the generated optical density at 600 nm (OD_600_) versus *t* growth curve (Fig. 1, left panels) is utilized to define the related area under the curve, $A_{x_{m}}$, as shown in equation 2:

(2) $$A_{x_{m}}=\overset{t_{f}}{\underset{t_{i}}{\int }}{\mathrm{OD}}_{600}\left(x_{m}\right)\;\mathrm{dt}$$

The Growth Reduction Coefficient of phage *x* at MOI *m*, ${\mathrm{GRC}}_{x_{m}}$ (designated the *local* GRC at MOI *m*, in keeping with the terminology devised by Storms et al.), is then defined through comparison with the area under the growth curve of the untreated bacterial strain, $A_{0}$, as shown in equation 3:

(3) $${\mathrm{GRC}}_{x_{m}}=1-\frac{A_{x_{m}}}{A_{0}}$$

The GRC is thus expressed as a unitless value which can range up to 1, which represents complete abolition of bacterial growth; positive values near 0 reflect a poor ability to reduce bacterial growth, and negative values conversely suggest the tendency to improve bacterial growth. ${\mathrm{GRC}}_{x_{m}}$ values can thus be calculated for a range of relevant MOIs, and can subsequently be used to construct a ${\mathrm{GRC}}_{x_{m}}$ versus log_10_MOI curve (Fig. 1, right panels). The area under this curve, designated *A_x_*, is defined using equation 4:

(4) $$A_{x}=\overset{\mathrm{MAX MOI}}{\underset{\mathrm{MIN MOI}}{\int }}{\mathrm{GRC}}_{x_{m}}d(\log_{10}\mathrm{MOI})$$

The maximum theoretical area under this curve, designated *A*_MAX_, is then computed using the number of MOI conditions utilized, *n_m_*, as shown in equation 5:

(5) $$A_{\mathrm{MAX}}=n_{m}\;-\;1$$

The GRC of phage *x* across all tested MOIs, GRC*_x_* (designated the *global* GRC of phage *x*), is then defined as the quotient of *A_x_* and *A*_MAX_, as shown in equation 6:

(6) $${\mathrm{GRC}}_{x}=\frac{A_{x}}{A_{\mathrm{MAX}}}$$

GRC*_x_* and ${\mathrm{GRC}}_{x_{m}}$ values can subsequently be compared between the same phage on different hosts, or between different phages on the same host, to quantify the potential therapeutic suitability of various phages. A ${\mathrm{GRC}}_{x_{m}}$ value serves as a quantitative estimate of the antibacterial effect a given phage *x* has at a given MOI *m*, and these values can therefore be used to determine the optimal MOI for therapeutic applications; while the GRC*_x_* value provides a quantitative estimate of the antibacterial effect a phage has across a range of MOIs, and therefore serves as an indicator of the overall therapeutic usefulness of a phage. A phage which has a high ${\mathrm{GRC}}_{x_{m}}$ at only a single MOI has lower overall usefulness than a phage which has high ${\mathrm{GRC}}_{x_{m}}$ across the entire MOI range, and this is reflected in the relative GRC*_x_* values of the two phages.

**FIG 2:**
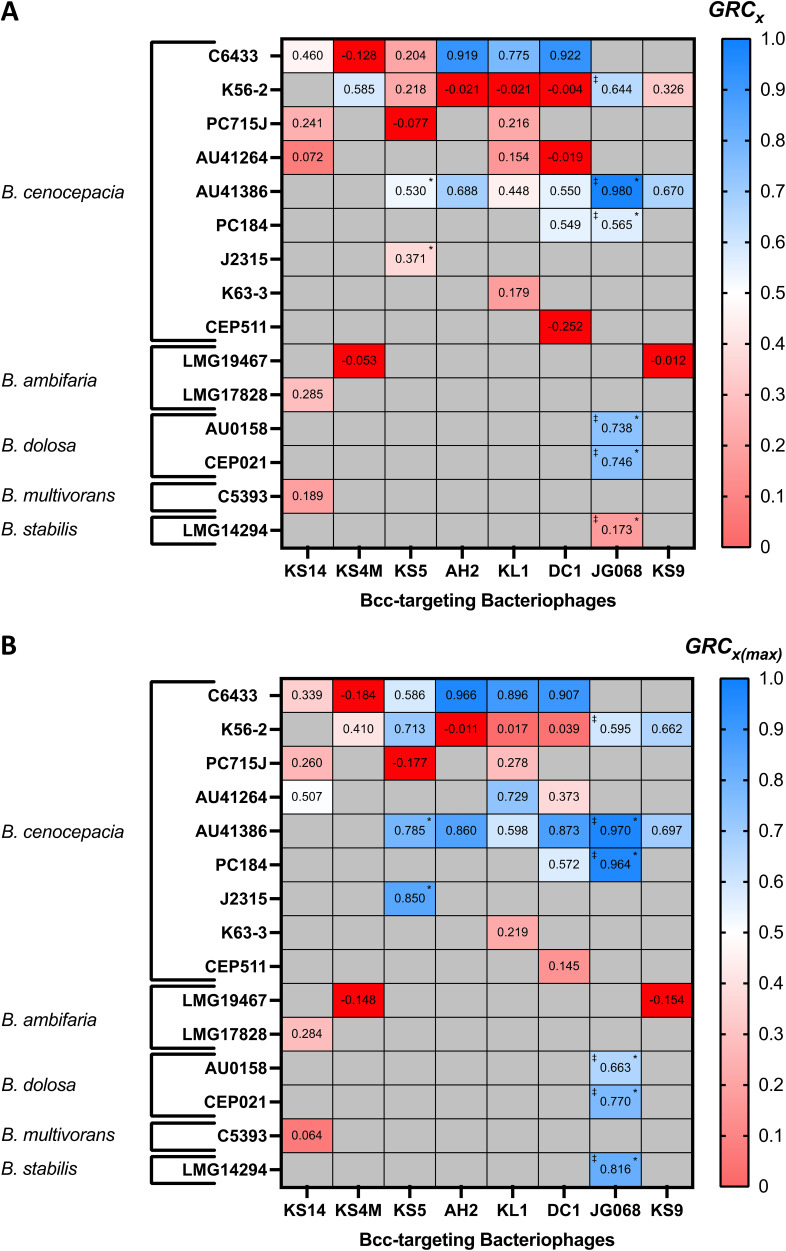
GRC of all available Bcc-phage host pairs. All infections were conducted under standard conditions across the standard MOI range of 0.1 to 10,000, except for cells designated with an asterisk (*), which indicate phage-host pairs for which the maximum available MOI was 1,000; here, GRC*_x_* (A) and ${\mathrm{GRC}}_{x_{\max}}$ (B) values were then computed for each phage-host pair using equations 6 and 3, respectively. Data are presented as heatmaps with high positive GRC values in blue, low positive GRC values in pink, and negative values in red, and GRC values are also provided numerically in each cell. Cells designated with stacked dagger symbols (‡) indicate phage-host pairs containing the OL phage JG068, while cells shaded in gray represent phage-host pairs which did not satisfy the EPA threshold and were not investigated. Values represent the mean of at least three biological replicates.

GRC experiments of this type were conducted for all Bcc phage-host pairs for which an EPA of ≥−5 was obtained (*n* = 37; representative data in Fig. 1; all data in Fig. S2). When investigating Bcc phages which had positive GRC*_x_*, implying that they have at least some antibacterial activity, we found that overall, the ${\mathrm{GRC}}_{x_{m}}$ increases with the MOI (Fig. S3). A similar relationship was identified mathematically between MOI and VI for Escherichia coli phages T4, T5, and T7 under all tested conditions, as well as between the MOI and qualitatively assessed growth curve reduction for Vibrio anguillarum phage VP-2, Enterococcus faecalis phage EFDG1, Klebsiella pneumoniae phage ZCKP1, and Acinetobacter baumannii phage KARL-1, possibly suggesting a widespread direct relationship between phage MOI and antibacterial activity (22, 48–51). Interestingly, the optimal MOI ranges of most Bcc phages appear to be considerably higher than those reported for phages infecting some bacterial species (22, 48, 50) yet not others (49, 51), but the mechanistic causes behind this remain difficult to elucidate. Since this finding suggests that Bcc phages could be useful at high MOIs, even if their overall effectiveness across all MOIs is low, we considered both the GRC*_x_* and the ${\mathrm{GRC}}_{x_{m}}$ at the maximum available MOI, designated ${\mathrm{GRC}}_{x_{\max}}$, when evaluating the potential therapeutic usefulness of Bcc phages. To establish a guideline by which to determine whether Bcc phages could have therapeutic potential, we set a GRC threshold of GRC*_x_ or* ${\mathrm{GRC}}_{x_{\max}}$ = 0.05, meaning that a phage must reduce bacterial growth by at least 5% through either of these metrics to be considered therapeutically useful in the context of this exploratory study. Values for GRC*_x_* and ${\mathrm{GRC}}_{x_{\max}}$ are summarized for all tested phage-host pairs in Fig. 2, demonstrating substantial variability for both metrics among Bcc-targeting phages.

Of the 37 phage-host pairs investigated, 7 failed to pass the GRC threshold since neither their GRC*_x_* nor ${\mathrm{GRC}}_{x_{\max}}$ values reached 0.05 (representative example: phage KS4M on B. cenocepacia strain C6433; Fig. 1A). Interestingly, all 7 of these pairs had a negative GRC*_x_*, suggesting that these phages increase bacterial growth rather than reducing it. This phenomenon was not seen under any conditions for the phages investigated using the VI (22), implying that these are the first examples of phages being mathematically demonstrated to improve bacterial growth. Among the 30 remaining pairs, three distinct MOI-${\mathrm{GRC}}_{x_{m}}$ trends were identified. A direct relationship between these variables, as described previously and in other studies (22, 48, 49), was seen in 16 of these pairs (representative example: phage KL1 on B. cenocepacia strain AU41264; Fig. 1B), while a modest inverse relationship was observed for only 2 pairs (representative example: phage KS14 on B. multivorans strain C5393; Fig. 1C). Interestingly, in 6 of the pairs, the ${\mathrm{GRC}}_{x_{m}}$ of the phage did not change significantly across the tested MOI range, suggesting that in these cases the GRC is independent of the MOI (representative example: KS9 on B. cenocepacia strain AU41386; Fig. 1D). In the remaining 6 pairs, the relationships between MOI and ${\mathrm{GRC}}_{x_{m}}$ were irregular, fitting into none of the patterns identified above. In some of these cases, such as phage KL1 on B. cenocepacia strain C6433 (see Fig. S2C), the overall MOI-${\mathrm{GRC}}_{x_{m}}$ trend was positive, but unusual features—such as vertices at intermediate MOIs—prevented their classification into the relevant category. Phages exhibit highly distinct MOI-${\mathrm{GRC}}_{x_{m}}$ trends (see Fig. S3), in addition to highly distinct ${\mathrm{GRC}}_{x_{m}}$ values, on different bacterial strains, indicating that the specific phage-host pair is crucial to whether a phage may be therapeutically effective. These highly variable findings highlight the enormous diversity in the antimicrobial effects of both LC and OL Bcc phages and underscore the need to evaluate these phages against particular hosts on a case-by-case basis. Moreover, we found that EPA is a poor predictor of the antibacterial effects of phages against planktonic cells as measured by GRC (Fig. S4). Specifically, although modest correlations were observed between EPA and both GRC*_x_* and ${\mathrm{GRC}}_{x_{\max}}$ (see Table S3), and a low EPA is reasonably predictive of low GRC*_x_* and ${\mathrm{GRC}}_{x_{\max}}$, a high EPA is by no means predictive of high GRC*_x_* or ${\mathrm{GRC}}_{x_{\max}}$. As a result, although a low EPA may be used to disqualify certain phage-host pairs from further experimentation, the opposite is not true because a high EPA does not automatically suggest that the phage might be effective against that specific host in liquid medium, or possibly *in vivo*.

### The Endpoint Growth Reduction Coefficient inaccurately approximates the Growth Reduction Coefficient.

Approaches such as the VI or GRC, which use data for growth over an extended period of time, are obviously more informative than approaches which use data from the individual endpoint of that growth period because they contain more data and therefore provide a more accurate depiction of the situation as a whole. These techniques, however, are either costly or time-consuming depending on whether or not modern instruments are available, and using an endpoint measurement as an approximation may therefore be tempting (49, 52). To investigate whether such an approximation might be valid, we defined an endpoint Growth Reduction Coefficient (eGRC), which is similar to the GRC but uses a single endpoint growth measurement rather than the area under a continuous growth curve. In this approach, a target bacterial strain is grown in the presence and absence of a phage *x* at various MOIs (*m*), and we utilize the difference between the final and initial growth values to define the endpoint Growth, ${\mathrm{eG}}_{x_{m}}$, as shown in equation 7:

(7) $${\mathrm{eG}}_{x_{m}}={\mathrm{OD}}_{600_{t\left(f\right)}}\left(x_{m}\right)\;-\;{\mathrm{OD}}_{600_{t\left(i\right)}}\left(x_{m}\right)$$

The endpoint Growth Reduction Coefficient of phage *x* at MOI *m*, ${\mathrm{eGRC}}_{x_{m}}$, is then defined through comparison with the endpoint growth of the untreated bacterial strain, eG_0_, as shown in equation 8:

(8) $${\mathrm{eGRC}}_{x_{m}}=1\;-\;\frac{{\mathrm{eG}}_{x_{m}}\;}{{\mathrm{eG}}_{0}}$$

**FIG 3:**
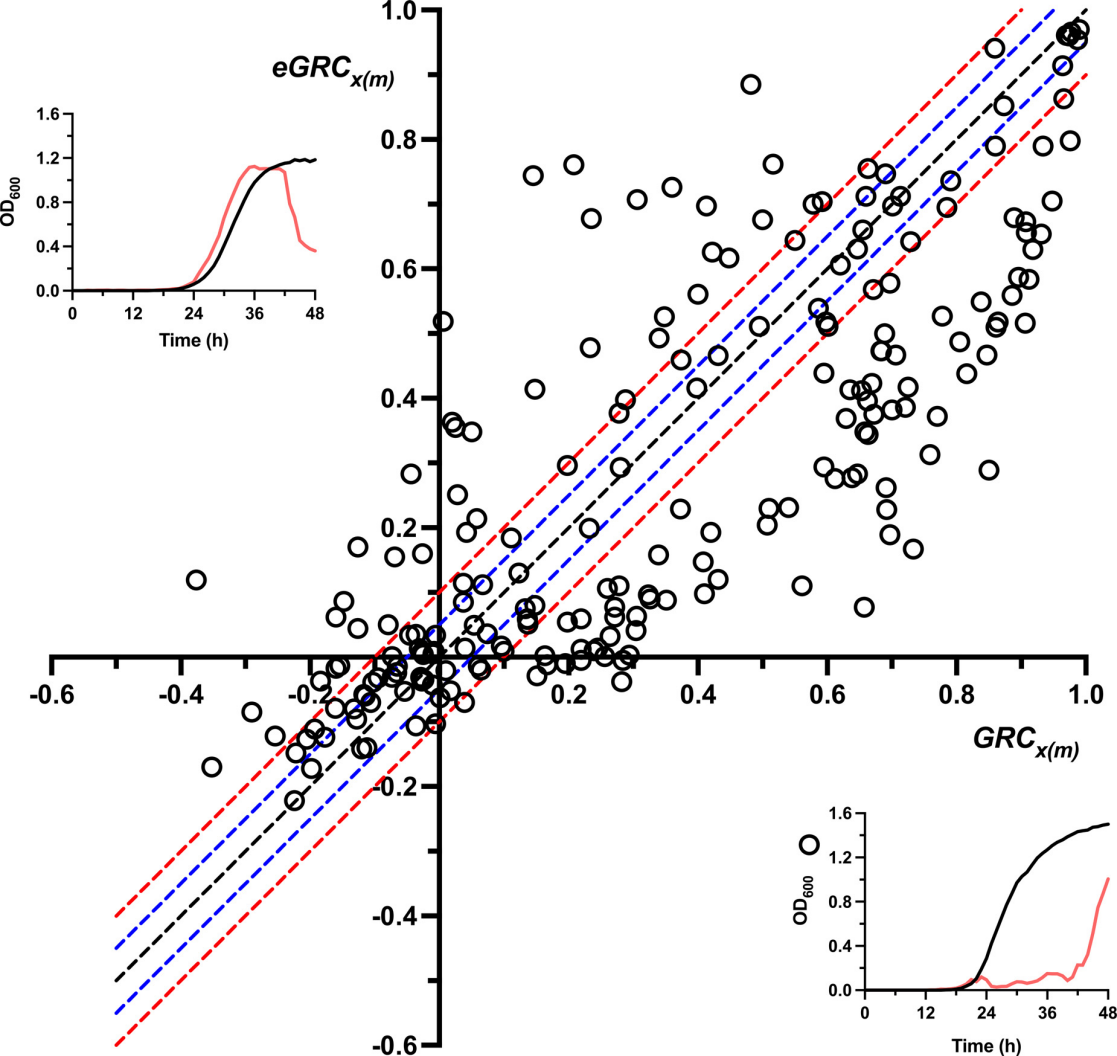
Comparison of ${\mathrm{GRC}}_{x_{\max}}$ and ${\mathrm{eGRC}}_{x_{m}}$ of phages targeting the Bcc. ${\mathrm{GRC}}_{x_{\max}}$ and ${\mathrm{eGRC}}_{x_{m}}$ values of all investigated Bcc phage-host pairs, for infections conducted under standard conditions across the standard MOI range of 0.1 to 10,000, were calculated using equations 3 and 8, respectively, and plotted using an *x*-*y* scatterplot. The absolute difference between the two values was computed for each phage-host pair and was used to determine the accuracy with which ${\mathrm{eGRC}}_{x_{m}}$ could estimate the ${\mathrm{GRC}}_{x_{\max}}$ across this infection period. If ${\mathrm{eGRC}}_{x_{m}}$ were a perfect predictor of ${\mathrm{GRC}}_{x_{\max}}$, all plotted points should fall on the black dashed mirror line, which represents identical ${\mathrm{eGRC}}_{x_{m}}$ and ${\mathrm{GRC}}_{x_{\max}}$ values. Points which fall above the mirror line are cases in which the ${\mathrm{eGRC}}_{x_{m}}$ overestimates the ${\mathrm{GRC}}_{x_{\max}}$, while those below the mirror line are cases in which the ${\mathrm{eGRC}}_{x_{m}}$ underestimates the ${\mathrm{GRC}}_{x_{\max}}$. Blue and red dashed lines denote absolute ${\mathrm{GRC}}_{x_{\max}}$ – ${\mathrm{eGRC}}_{x_{m}}$ differences of 0.05 (5% of GRC) and 0.10 (10% of GRC), respectively: points between the blue lines (inclusive) have an absolute ${\mathrm{GRC}}_{x_{\max}}$ – ${\mathrm{eGRC}}_{x_{m}}$ difference of 0.05 or less, while points between the red lines (inclusive) have an absolute ${\mathrm{GRC}}_{x_{\max}}$ – ${\mathrm{eGRC}}_{x_{m}}$ difference of 0.10 or less. Inset panels on the top left and bottom right graphically depict theoretical situations in which the ${\mathrm{eGRC}}_{x_{m}}$ overestimates and underestimates the ${\mathrm{GRC}}_{x_{\max}}$, respectively, with the phage-treated and untreated growth curves appearing as red and black lines, respectively. Plotted points represent the mean values of at least three biological replicates.

Using this approach, we computed ${\mathrm{eGRC}}_{x_{m}}$ values at all available MOIs for all Bcc phage-host pairs for which EPA ≥ 10^−5^ was obtained and plotted them against their cognate ${\mathrm{GRC}}_{x_{m}}$ values to determine the similarity between the two values (Fig. 3). We found that the absolute difference between the ${\mathrm{GRC}}_{x_{m}}$ and ${\mathrm{eGRC}}_{x_{m}}$ values was 0.10 or less (≤10% of GRC metric) in only 40.93% of cases, meaning there was a substantial difference between these two values in the majority of cases. Among the plotted points for which ${\mathrm{GRC}}_{x_{m}}$≥ 0.05, meaning they could be considered therapeutically relevant according to our criteria, the absolute difference between the ${\mathrm{GRC}}_{x_{m}}$ and ${\mathrm{eGRC}}_{x_{m}}$ was 0.10 or less in only 30.26% of cases. In 13.82% of cases, the ${\mathrm{eGRC}}_{x_{m}}$ was substantially larger than the ${\mathrm{GRC}}_{x_{m}}$, while in the remaining 55.92% of cases the ${\mathrm{eGRC}}_{x_{m}}$ was substantially smaller than the ${\mathrm{GRC}}_{x_{m}}$. These findings suggest that in general, endpoint measurements such as ${\mathrm{eGRC}}_{x_{m}}$ provide inaccurate approximations of growth-curve based approaches like ${\mathrm{GRC}}_{x_{m}}$ because they severely underestimate the true growth reduction capabilities of phages, and should therefore be used only sparingly in situations where growth-curve based approaches are impossible.

**Stable lysogenization frequency, a novel metric for quantification of a phage’s tendency to form stable lysogens, reveals enormous host and environment-driven diversity in the stable lysogen formation behaviors of Bcc phages.** When considering the lysogenization behavior of an LC phage in the context of its therapeutic suitability, the tendency of the phage to form unstable, short-lived lysogens is irrelevant since these lysogens will quickly be destroyed upon the phage’s induction to the lytic cycle. Therefore, the important factor is the frequency with which the phage forms stable, long-term lysogens which contribute to bacterial outgrowth, which we call the stable lysogenization frequency [*f*_(s.lys)_]. Concordantly, we define *f*_(s.lys)_ as the proportion of survivors of infection with phage *x* (*s_x_*) which are lysogens of phage *x* (*l_x_*) after an extended period of time (*t*), at a particular set of environmental conditions, including MOI (*m*), infection medium (*M*), temperature (*T*), and the host bacterium (*h*), as shown in equation 9:

(9) $$f_{\left(s\mathrm{.lys}\right)}{\left[x\right]}_{{\left(m,M,T,h\right)}_{t}}=\frac{l_{x}}{s_{x}}$$

**FIG 4:**
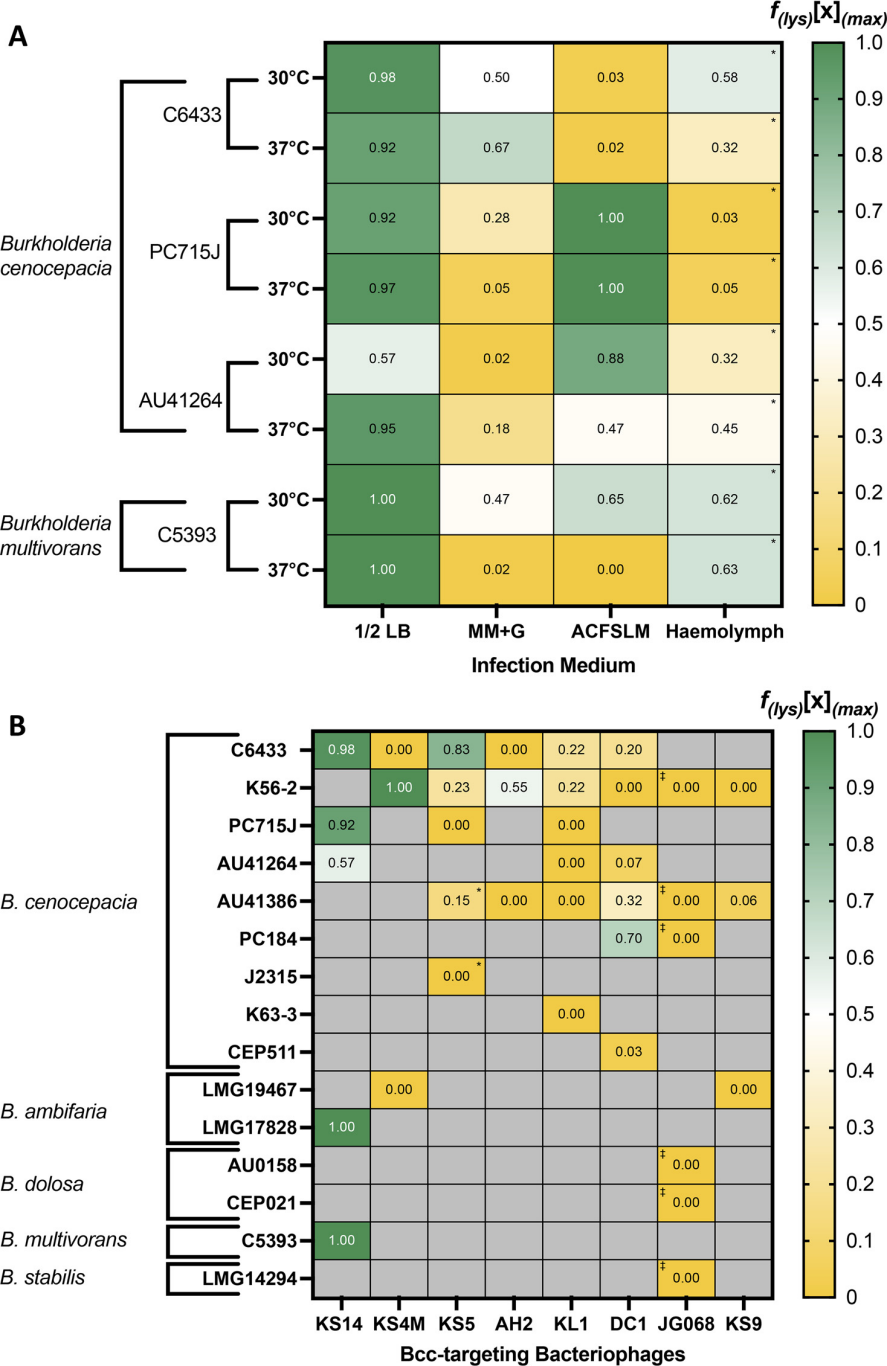
Stable lysogenization frequencies [*f*_(s.lys)_] of Bcc-targeting phages. Infections were conducted in different experimental media and *in vivo* in the hemolymph of Galleria mellonella larvae, at different temperatures and on different hosts with the Bcc-targeting myovirus KS14 (A), and under standard conditions for all Bcc phage-host pairs (B). All infections were performed at the maximum available MOI of 10,000, except for cells designated with an asterisk (*) which indicate phage-host pairs for which the maximum available MOI was 1,000, and *f*_(s.lys)_ values were then computed using equation 9. *f*_(s.lys)_ values for phage JG068 (B) were assigned automatically because JG068 is obligately lytic (OL) and genetically incapable of forming lysogens (cells designated with stacked dagger symbols, ‡). Data are presented as heatmaps with high *f*_(s.lys)_ values in green and low *f*_(s.lys)_ in yellow, and *f*_(s.lys)_ values are also provided numerically in each cell. Cells shaded in gray represent phage-host pairs which did not satisfy the EPA threshold and were not investigated. Values represent the mean of at least four biological replicates with 15 colonies tested for each biological replicate.

Since it can be demonstrated using the Poisson distribution that every bacterium is infected by at least one phage in an infection at any MOI ≥ 8 (53), every bacterium recovered from an infection at this MOI is by definition a survivor of infection, and the *f*_(s.lys)_ can therefore be estimated by screening these survivors for lysogeny. To investigate the effects of the infection medium, temperature, and the host organism on the *f*_(s.lys)_ of an LC phage, we quantified the *f*_(s.lys)_ of the myovirus KS14 in 48-h liquid infections at the maximum available MOI [(*f*_(s.lys)_(KS14)_(max)_], on three strains of B. cenocepacia and one strain of B. multivorans, in several different infection media at various temperatures (Fig. 4A). KS14 was selected for this experiment because it is the only phage in our panel which lysogenizes by forming a phagemid rather than by integrating into the host genome (8, 12) (Table S2), making it the least likely to be affected by phage-host *attP*/*attB* site incompatibility, and we therefore expected that the effects of environmental variables such as temperature and medium would be most apparent when working with this phage. Interestingly, *f*_(s.lys)_ was consistently high in half-strength Luria-Bertani (1/2 LB) medium at most of the tested temperature and host combinations but varied substantially with both host and temperature on other media. Simultaneously, M9 MM is associated with lower *f*_(s.lys)_, possibly suggesting that lysogeny is favored at higher nutrient densities among some Bcc phages. The *f*_(s.lys)_ of KS14 in Galleria mellonella hemolymph varies substantially with the infection temperature and bacterial host strain, suggesting that temperature-dependent factors, such as the rate of phage particle inactivation by hemolymph components (54, 55), relative growth rates of the wild-type and lysogenized bacterial strain, and relative stability of KS14 in its particle and phagemid form, may all interact and contribute to the stable lysogenization frequencies observed under these conditions. Although the individual and combined effects of these environmental variables on *f*_(s.lys)_ are undoubtedly complex and a mechanistic understanding of them remains elusive, particularly for the therapeutically relevant ACFSM and G. mellonella hemolymph, the observed diversity in *f*_(s.lys)_, even for a single phage, clearly demonstrates that the tendency of a phage to form stable lysogens is not determined solely by its genetic ability to do so. The *f*_(s.lys)_ should therefore be determined under relevant environmental conditions for any LC phage as part of its characterization and the determination of its suitability for therapeutic applications. Concordantly, we estimated the *f*_(s.lys)_ of all 8 Bcc phages on all available hosts, under the environmental conditions used in our GRC experiments, at the maximum available MOI (*f*_(s.lys)_[*x*]_(max)_). Considerable variability in *f*_(s.lys)_ was observed between different Bcc phages as well as within individual phages, depending on the bacterial host (Fig. 4B), further highlighting the importance of the specific phage-host pair to the phage’s tendency to form stable lysogens. Interestingly, many phage-host pairs produced no evidence of stable lysogeny despite the fact that all of these phages (except OL phage JG068) are genetically capable of forming lysogens. In some cases, this may be due to phage-host genetic incompatibility which prevents the integration of the phage genome into that of the host, while some strains may have other properties which prevent the establishment of lysogeny or cause rapid induction of the prophage to the lytic cycle. Although the mechanisms behind these findings remain elusive, it is clear that the stable lysogen-forming behaviors of these LC phages depend greatly on the particular strain being targeted, implying that at least some LC phages could potentially be used therapeutically against specific strains without treatment failure driven by lysogen formation.

### Low-*f*_(s.lys)_ LC Bcc phages can be highly effective at reducing bacterial growth.

**FIG 5:**
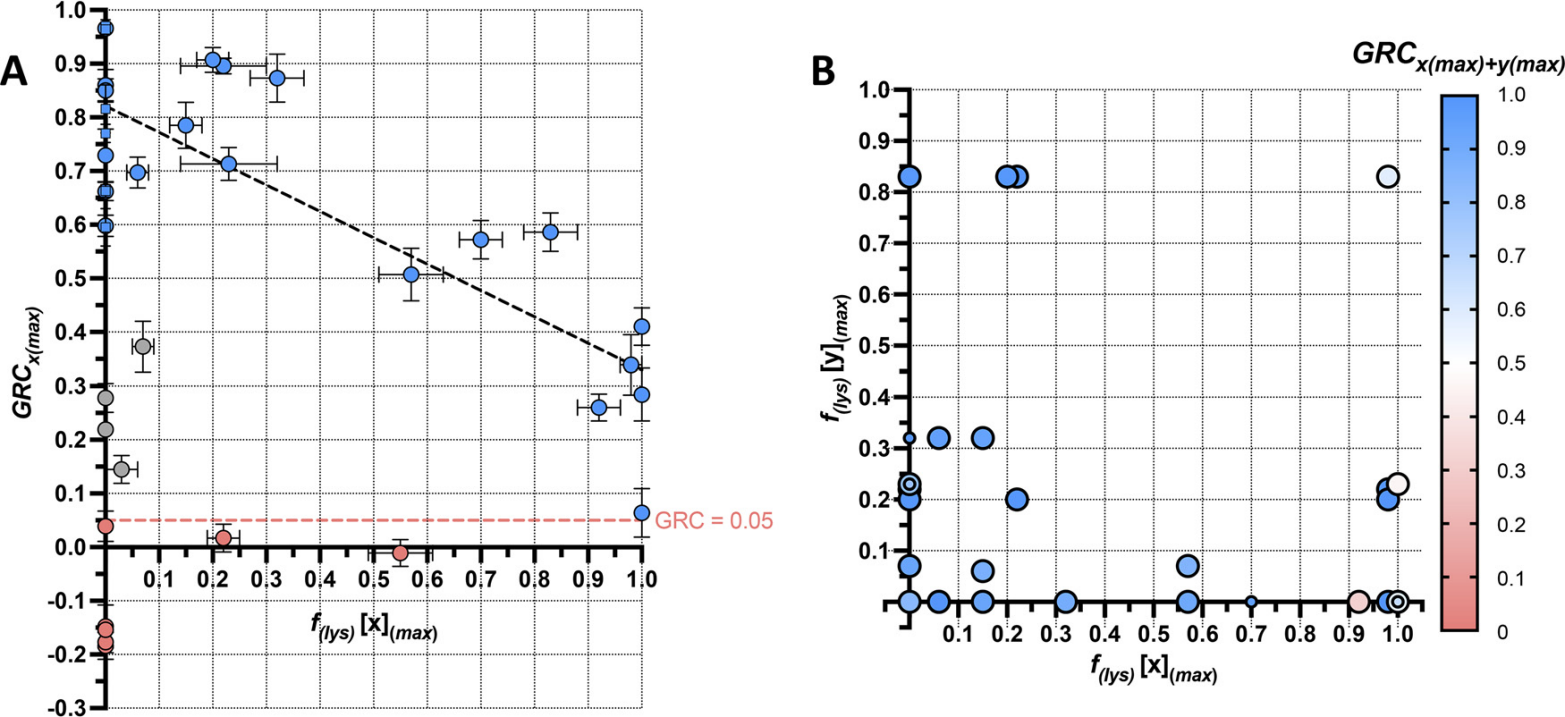
GRC as a function of stable lysogenization frequency [*f*_(s.lys)_]. (A) Correlations between ${\mathrm{GRC}}_{x_{m}}$ and *f*_(s.lys)_ at the maximum available MOI. ${\mathrm{GRC}}_{x_{m}}$ and *f*_(s.lys)_ values of all investigated Bcc single-phage-host pairs, for infections conducted under standard conditions at the maximum available MOI, were calculated using equations 3 and 9, respectively, and plotted using an *x*-*y* scatterplot. Red points represent phage-host pairs which did not satisfy the GRC ≥ 0.05 criterion and thus fall below the red dashed line (representing a GRC of precisely 0.05). Gray points represent phage-host pairs which did satisfy the GRC ≥ 0.05 criterion but formed an outlier group and have low ${\mathrm{GRC}}_{x_{m}}$ despite also having low *f*_(s.lys)_ values. Blue points represent phage-host pairs which satisfied the GRC ≥ 0.05 criterion and followed the overall trend of ${\mathrm{GRC}}_{x_{m}}$ and *f*_(s.lys)_ values being inversely correlated. Circles and squares represent phage-host pairs containing lysogenization-capable (LC) and OL phages, respectively. Black dashed line represents the line-of-best-fit for blue points. Vertical and horizontal error bars represent SEM of at least three and four biological replicates, respectively. (B) ${\mathrm{GRC}}_{x_{m}+y_{m}}$ of two-phage cocktail-host pairs as a function of the *f*_(s.lys)_ values of the constituent phages of the cocktail, at the maximum available MOI. ${\mathrm{GRC}}_{x_{m}+y_{m}}$ values of all possible 1:1-ratio two-phage cocktail-host pairs, along with the *f*_(s.lys)_ values of the constituent phages, were computed using equations 3 and 9, respectively, for infections conducted under standard conditions at the maximum available MOI. Combinations containing two LC phages are shown in large bubbles, while combinations containing the OL phage JG068 are shown in smaller bubbles (in some cases superimposed onto larger bubbles). These values are plotted using a two-dimensional scatterplot on which a third dimension, color, is used to represent ${\mathrm{GRC}}_{x_{\max}+y_{\max}}$. Plotted values represent the mean of at least three and four biological replicates for ${\mathrm{GRC}}_{x_{m}+y_{m}}$ and *f*_(s.lys)_, respectively.

Because many LC Bcc phages have low *f*_(s.lys)_ and may therefore engage in replication behavior similar to that of OL phages, we sought to investigate whether these phages also have high growth reduction capabilities and might therefore be therapeutically useful. Concordantly, we compared the ${\mathrm{GRC}}_{x_{\max}}$ (Fig. 2B) and *f*_(s.lys)_[*x*]_(max)_ (Fig. 4B) of all tested Bcc phage-host pairs (*n* = 37) and found a strong inverse correlation between these variables among phage-host pairs for which at least modest antibacterial activity was observed (Fig. 5A). A similar comparison between *f*_(s.lys)_[*x*]_(max)_ (Fig. 4B) and GRC*_x_* (Fig. 2A) found no significant correlation (Fig. S5; see Table S3 for details), which is unsurprising because *f*_(s.lys)_ likely varies with the MOI. As previously described, 7 of these phage-host pairs failed to meet the established GRC ≥ 0.05 criterion, and no significant relationship between *f*_(s.lys)_[*x*]_(max)_ and ${\mathrm{GRC}}_{x_{\max}}$ was identified for these pairs (Fig. 5A, see Table S3 for details). Of the remaining 30 phage-host pairs, 26 (87%) followed a strong overall trend of *f*_(s.lys)_[*x*]_(max)_ and ${\mathrm{GRC}}_{x_{\max}}$ being inversely correlated (R^2^ = 0.67; *P* < 0.0001), while another 4 pairs (13%) simultaneously had low *f*_(s.lys)_[*x*]_(max)_ and ${\mathrm{GRC}}_{x_{\max}}$ and thus formed a small outlier group. Even when this outlier group is included, the inverse correlation between *f*_(s.lys)_[*x*]_(max)_ and ${\mathrm{GRC}}_{x_{\max}}$ remains strong (R^2^ = 0.27; *P = *0.0033). Indeed, among the 30 phage-host pairs which satisfy the GRC ≥ 0.05 criterion, all of those with *f*_(s.lys)_[*x*]_(max)_ > 0.4 have ${\mathrm{GRC}}_{x_{\max}}$< 0.6, while 83% of those with a *f*_(s.lys)_[*x*]_(max)_ < 0.4 have a ${\mathrm{GRC}}_{x_{\max}}$< 0.59, meaning that *f*_(s.lys)_[*x*]_(max)_ is a fairly powerful predictor of ${\mathrm{GRC}}_{x_{\max}}$ among these phages (Fig. 5A). The presence of the outlier group suggests that a low (or absent) tendency to form stable lysogens does not invariably imply that a phage is effective at reducing growth, a finding further supported by the fact that 6 of the 7 phage-host pairs which failed to meet the GRC ≥ 0.05 criterion also had low *f*_(s.lys)_[*x*]_(max)_. Importantly, this reiterates the fact that lysogeny is not the only obstacle to finding phages with strong antibacterial activity against certain host strains. Nevertheless, the fact that the vast majority of potentially therapeutically useful (GRC ≥ 0.05) Bcc phages follow a strong inverse correlation between *f*_(s.lys)_[*x*]_(max)_ and ${\mathrm{GRC}}_{x_{\max}}$ implies that high-*f*_(s.lys)_[*x*]_(max)_ phages tend to have relatively low ${\mathrm{GRC}}_{x_{\max}}$ and are therefore unsuitable for monophage therapy, while low-*f*_(s.lys)_[*x*]_(max)_ phages generally tend to have relatively high ${\mathrm{GRC}}_{x_{\max}}$ and could therefore be therapeutically useful. Crucially, several low-*f*_(s.lys)_[*x*]_(max)_ LC phage-host pairs, such as those including LC phages AH2, KL1, or DC1, have ${\mathrm{GRC}}_{x_{\max}}$ values comparable to (and in some cases higher than) those seen with the OL phage JG068 (Fig. 5A), strongly suggesting that low-*f*_(s.lys)_[*x*]_(max)_ LC phages such as these may be suitable for monophage therapy.

**FIG 6:**
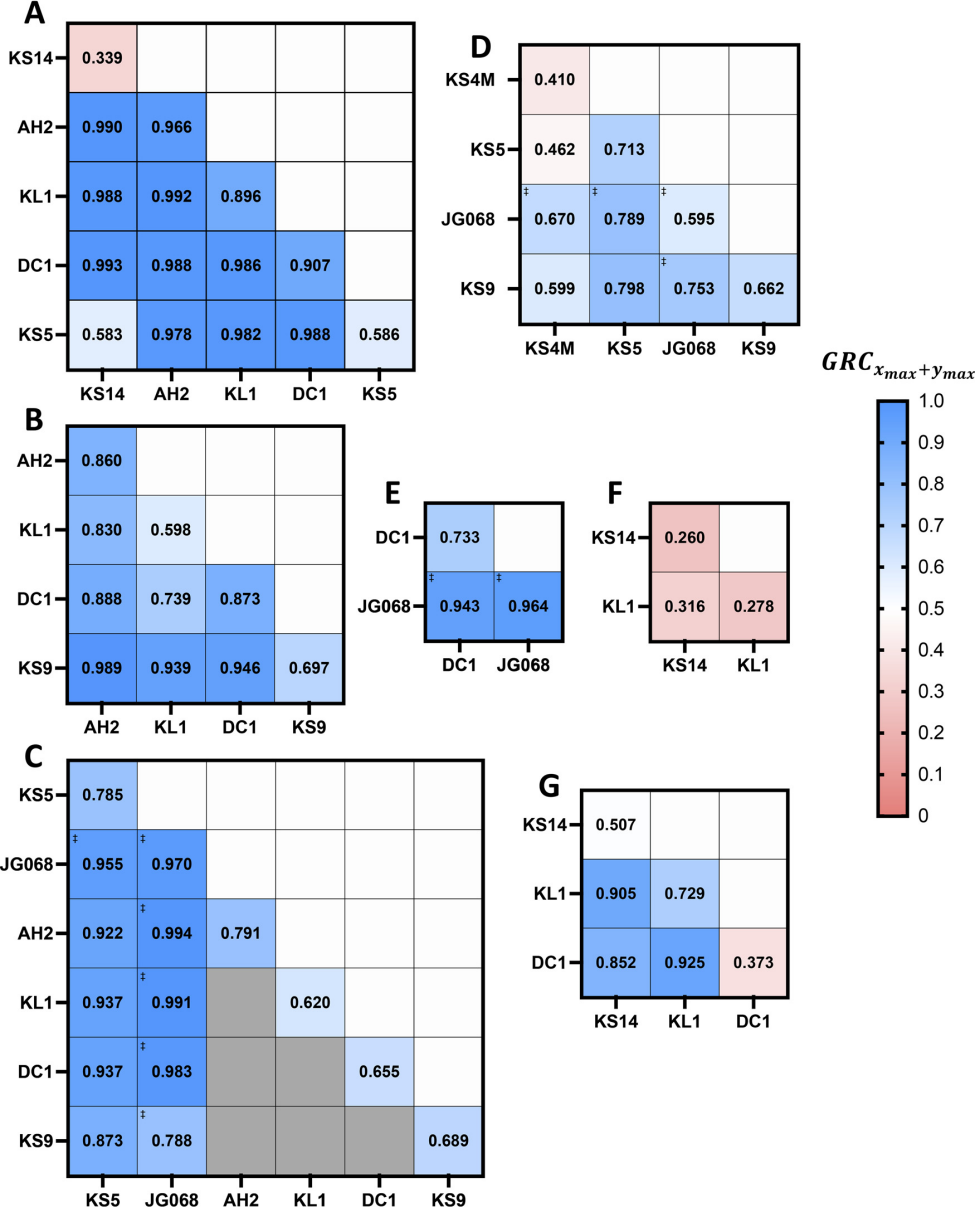
GRC at maximum available MOI for all available Bcc single-phage-host and two-phage cocktail-host pairs. ${\mathrm{GRC}}_{x_{m}}$ values for all Bcc single-phage-host pairs, as well as ${\mathrm{GRC}}_{x_{m}+y_{m}}$ values for all possible 1:1-ratio two-phage cocktail-host pairs, for infections conducted under standard conditions at the maximum available MOI, were calculated using equation 3. Data are shown as heatmaps for all host strains for which more than one potentially therapeutically useful (GRC ≥ 0.05) phage is available, including B. cenocepacia strains C6433 (A), AU41386 at MOI = 10^4^ (B) and MOI = 10^3^ (C), K56-2 (D), PC184 (E), PC715J (F), and AU41264 (G). Cells designated with stacked dagger symbols (‡) indicate single-phage-host pairs and two-phage cocktail-host pairs containing the OL phage JG068. ${\mathrm{GRC}}_{x_{m}}$ and ${\mathrm{GRC}}_{x_{m}+y_{m}}$ values are color-coded within the heatmaps, with high GRC values in blue and low values in pink, and GRC values are also provided numerically in each box. Gray boxes in panel C represent pairs which were not investigated at an MOI of 10^3^ because the maximum possible MOI of these single-phage-host and thus two-phage cocktail-host pairs is 10^4^; these pairs are displayed in panel B. Values represent the mean of at least three biological replicates.

**High-*f*_(s.lys)_ LC Bcc phages can be highly effective at reducing bacterial growth when combined with low-*f*_(s.lys)_ LC or OL counterpart phages to form two-phage cocktails.** Since high-*f*_(s.lys)_ LC phages have low ${\mathrm{GRC}}_{x_{\max}}$ and are clearly unsuitable for therapeutic use on their own, we sought to investigate whether their therapeutic utility might be rehabilitated when they are combined with other phages. Concordantly, we constructed two-phage cocktails of all possible 1:1-ratio combinations of potentially therapeutically useful (GRC ≥ 0.05) Bcc phages and investigated their growth reduction effects, at the maximum possible MOI, on all B. cenocepacia strains for which more than one phage is available (Fig. 6). Although the antibacterial effects of the two-phage cocktails, measured using ${\mathrm{GRC}}_{x_{\max}+y_{\max}}$, were in many cases higher than the effects of either of the constituent phages on their own (${\mathrm{GRC}}_{x_{\max}}$ and ${\mathrm{GRC}}_{y_{\max}}$), this effect was dependent on both the specific combination of phages being used and the host strain being targeted. Among the cocktails of two low-*f*_(s.lys)_ LC phages and OL or low-*f*_(s.lys)_ LC phages, the magnitude of the improvement in antibacterial effect was highly variable, often because many of these phages are already highly effective at reducing bacterial growth on their own. The OL phage JG068, for instance, did not have substantially improved antibacterial activity when combined with any other phages targeting strains PC184 (Fig. 6E) or AU41386 (Fig. 6C), likely because JG068 is already extremely effective against both of these strains on its own. Conversely, the antibacterial effect of JG068 was slightly improved when it was combined with any other phage targeting strain K56-2 (Fig. 6D), for which its independent ${\mathrm{GRC}}_{x_{\max}}$ is lower. Similarly, the effectiveness of the low-*f*_(s.lys)_ LC phage AH2 was not significantly improved by combination with any phage against strains C6433 (Fig. 6A) or AU41386 (Fig. 6B) except for phage KS9, which substantially improved the antibacterial effect of AH2 on AU41386. Other low-*f*_(s.lys)_ LC phages such as DC1 and KL1 also followed this trend, improving each other’s effectiveness against certain strains, such as C6433 (Fig. 6A) and AU41264 (Fig. 6G), but not against AU41386 (Fig. 6B). Crucially, two-phage combinations containing a high *f*_(s.lys)_ LC phage paired with an OL or low-*f*_(s.lys)_ counterpart phage also have increased antibacterial effects relative to those of either constituent phage on its own. For instance, KS14, which has high *f*_(s.lys)_ on all susceptible strains, improves the antibacterial effectiveness of all low-*f*_(s.lys)_ phages with which it is paired against strains C6433 (Fig. 6A), PC715J (Fig. 6F), and AU41264 (Fig. 6G). Phage KS5 substantially improves the antibacterial activity of all low-*f*_(s.lys)_ phages on C6433 (Fig. 6A), against which it has a high *f*_(s.lys)_, but only modestly on K56-2 (Fig. 6D), against which it has a relatively low *f*_(s.lys)_. However, KS5 also substantially improves the effectiveness of all low-*f*_(s.lys)_ phages on AU41386 (Fig. 6C), against which it itself has low *f*_(s.lys)_, implying that the interactions between KS5 and other phages are, predictably, not affected solely by lysogenization. Nevertheless, these results demonstrate that high-*f*_(s.lys)_ LC phages remain therapeutically useful because they can substantially improve the antibacterial effects of OL or low-*f*_(s.lys)_ phages with which they are combined. This finding is also evident when visualizing the antibacterial effect of a combination (${\mathrm{GRC}}_{x_{\max}+y_{\max}}$) as a function of the *f*_(s.lys)_ of its constituent phages (Fig. 5B). Although high-*f*_(s.lys)_ LC phages are unsuitable for monophage therapy because they produce only limited growth reduction when employed on their own (Fig. 5A), cocktails in which such high-*f*_(s.lys)_ phages are combined with OL or low-*f*_(s.lys)_ LC counterpart phages consistently produce high levels of bacterial growth reduction (Fig. 5B). Only two combinations of high-*f*_(s.lys)_–low-*f*_(s.lys)_ phages (KS4M and KS5 on K56-2, Fig. 6B; KS14 and KL1 on PC715J; Fig. 6F) produce poor antibacterial effects, and the low effectiveness of the constituent phages may be responsible for this in both cases. Only one cocktail of two high-*f*_(s.lys)_ phages (KS14 and KS5 on C6433, Fig. 6A) was tested, and it was found to have a limited ability to reduce bacterial growth, suggesting that when used in cocktails, high-*f*_(s.lys)_ phages should be paired only with low-*f*_(s.lys)_ or OL phages to achieve the maximum antibacterial effect.

### LC Bcc phages interact synergistically to maximize reduction of bacterial growth.

While many of the two-phage combinations described above appear to have antibacterial effects greater than the individual effects of each of their constituent phages (Fig. 6), rigorous mathematical analysis is required to identify whether these differences are significant and whether they constitute synergistic interactions between phages or are merely the results of additive or independent effects. In studies investigating the effects of antibiotic combinations, independent or autonomous activity is defined, either qualitatively or quantitatively, as the antibacterial effect of combined treatment being indistinguishable from the effect of a single constituent of the combination; this suggests that this constituent is acting independently of the other agent and is responsible for most of the antimicrobial effect (56–59). Here, we use the GRC metric to mathematically define autonomous activity in a cocktail of two phages *x* and *y* at a concentration *m* as a situation in which the GRC of the cocktail is statistically indistinguishable from that of either constituent phage, as shown in equation 10:

(10) $${\mathrm{GRC}}_{x_{m}+y_{m}}={\mathrm{GRC}}_{x_{m}}{\;\mathrm{or}\;\mathrm{GRC}}_{y_{m}}$$

If an autonomous effect is not occurring, it can be surmised that both phages are contributing meaningfully to the antibacterial effect, and their effects must therefore be additive, synergistic, or antagonistic. If the antibacterial effects of the constituent phages are additive, we expect that the proportion of growth which remains after combined treatment should be statistically indistinguishable from the product of the proportions of growth which remain after treatment with each of the individual constituents (39, 59–61). Using the terms defined above, we can express this mathematically in the following equation:

$$\frac{A_{x_{m}+y_{m}}}{A_{0}}=\frac{\left(A_{x_{m}})(A_{y_{m}}\right)}{{\left(A_{0}\right)}^{2}}$$

Rewriting this expression in terms of GRC, we define an additive effect as shown in equation 11:

(11) $${\mathrm{GRC}}_{x_{m}+y_{m}}={\mathrm{GRC}}_{\left[\left(x_{m}\right)\left(y_{m}\right)\right]}$$

where the right-side term ${\mathrm{GRC}}_{\left[\left(x_{m}\right)\left(y_{m}\right)\right]}$ is used as a short form of $1-\frac{\left(A_{x_{m}})(A_{y_{m}}\right)}{{\left(A_{0}\right)}^{2}}$. If the antibacterial interaction of the phages is synergistic, we expect that the growth reduction effect of the combination is significantly greater than the product of the individual antibacterial effects of the constituent phages, and we express this mathematically using GRC as shown in equation 12:

(12) $${\mathrm{GRC}}_{x_{m}+y_{m}}>{\mathrm{GRC}}_{\left[\left(x_{m}\right)\left(y_{m}\right)\right]}$$

Conversely, it is possible that one of the phages has a much stronger antibacterial effect, but the second phage reduces the effectiveness of the first. In such cases, the antibacterial interaction between the phages is antagonistic, and we expect the growth reduction effect of the combination to be significantly less than the product of the individual antibacterial effects of the constituent phages (equation 13) and also significantly less than the growth reduction effect of at least the more effective of the two phages (equation 14):

(13) $${\mathrm{GRC}}_{x_{m}+y_{m}}\;<\;{\mathrm{GRC}}_{\left[\left(x_{m}\right)\left(y_{m}\right)\right]}$$

(14) $${\mathrm{GRC}}_{x_{m}}>{\mathrm{GRC}}_{x_{m}+y_{m}}>{\mathrm{GRC}}_{y_{m}}$$

**FIG 7:**
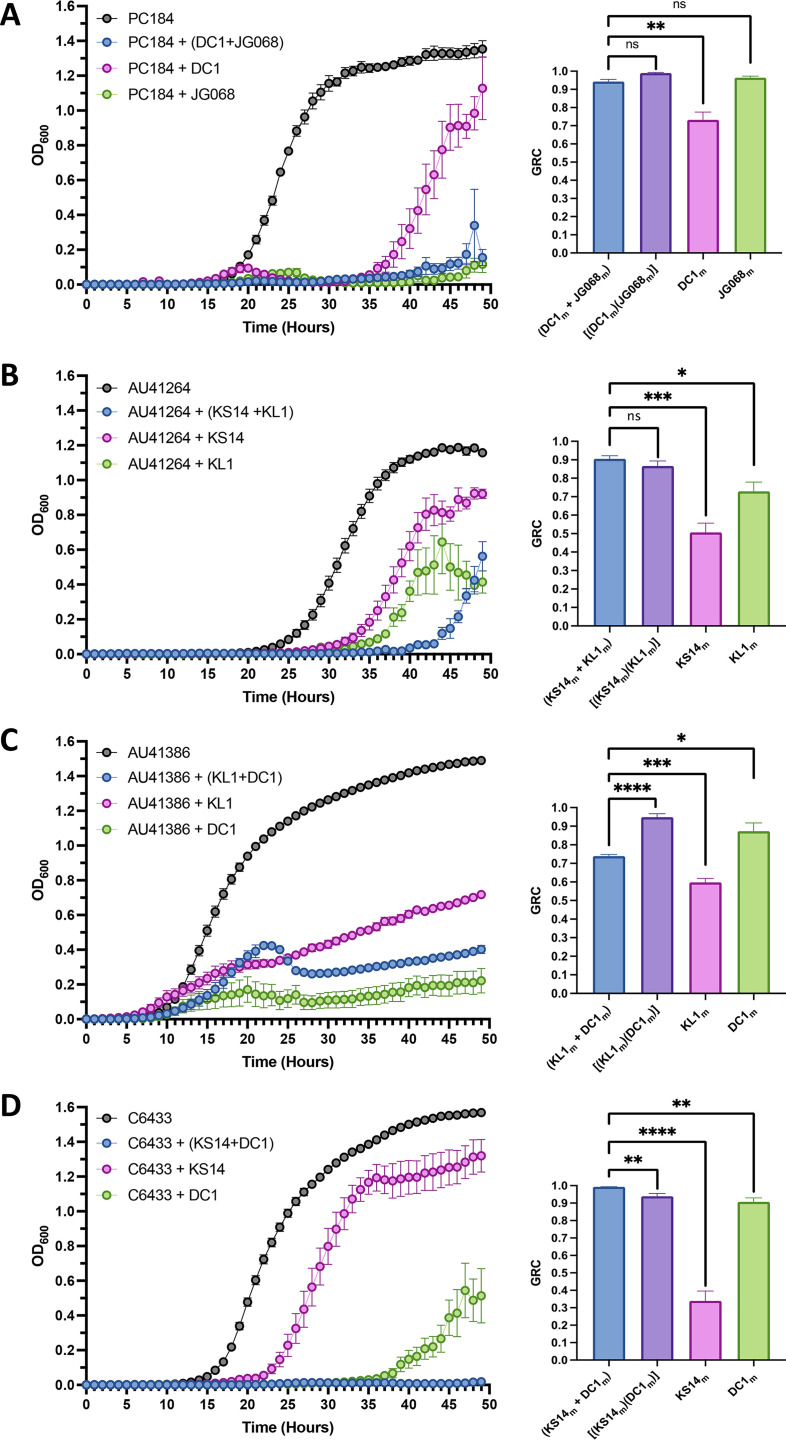
Representative interactions between Bcc-targeting phages. Bacterial growth curves (left) and treatment GRC bar charts (right) representative of two-phage combination-host pairs in which the phages behave (A) autonomously, where the effectiveness of the combination is statistically indistinguishable from that of at least one phage alone; (B) additively, where the effectiveness of the combination is statistically indistinguishable from the product of the individual efficacies of the component phages; (C) antagonistically, where the effectiveness of the combination is statistically less than the product of the individual efficacies of the component phages; or (D) synergistically, where the effectiveness of the combination is statistically greater than the product of the individual efficacies of each component phage. Purple bars represent the product of the individual efficacies of the component phages, as computed using the right-hand side of equation 11. Black line represents bacterial growth without phage. Blue lines and bars represent bacterial growth with and GRC of the phage pair; pink and green lines and bars represent bacterial growth with and GRC of each individual phage. Bars represent the SEM of at least three biological replicates. Statistically significant differences between groups were assessed using Student’s *t* tests (with Welch’s correction for unequal variances). ****, *P* < 0.0001; ***, *P* < 0.001; **, *P* < 0.01; *, *P* < 0.05; ns, *P* > 0.05.

We conducted this mathematical analysis for all two-phage combinations tested against susceptible strains (*n* = 36; see Fig. 6) and identified six distinct types of antibacterial effects among these combinations (representative data in Fig. 7, all data in Fig. S6, summarized in Table 1). Autonomous activity, defined in equation 10, was identified in 22 combinations (61.2%). In 18 (50.0%) of these, the overall antibacterial effect was driven by the more effective phage in the combination (representative example: JG068 and DC1 against PC184; Fig. 7A). Many of these combinations involve the OL phage JG068 and the low-*f*_(s.lys)_ LC phage AH2, which are each extremely effective at reducing bacterial growth on their own and therefore there is little room for improvement through combination with another phage. Combinations tested against strain K56-2 (Fig. 6D), which have modest and statistically insignificant improvements in antibacterial activity relative to their constituents, also fall into this category. In 2 (5.6%) combinations, the overall effect was driven by the less effective phage (Fig. S6P and Fig. S6AD), while the antibacterial effects of the other 2 (5.6%) combinations were statistically indistinguishable from the effects of either of the constituents, likely because the constituents of these combinations were equally effective and ineffective at reducing growth (Fig. S6E and Fig. S6AK, respectively). Additive effects, as defined in equation 11 (representative example: KS14 and KL1 on AU41264; Fig. 7B), were identified for 7 (19.4%) combinations, all of them involving LC phages, and only 1 (2.7%) combination, KL1 and DC1 on AU41386 (Fig. 7C), exhibited antagonistic effects as defined in equations 13 and 14. Synergistic interactions, defined in equation 12 (representative example: KS14 and DC1 on C6433; Fig. 7D), were identified for 6 (16.7%) tested combinations. Crucially, all 6 of these synergistic combinations were composed of LC phages and, interestingly, 4 of them contained one of the high-*f*_(s.lys)_ LC phages KS14 or KS5. Although the strong antibacterial effects of these synergistically interacting phages suggest potential therapeutic utility, phage preparations for *in vivo* use sometimes have lower MOIs than those used in these experiments, and we therefore sought to explore whether these synergistic interactions are preserved at lower MOIs. Concordantly, we tested our 6 synergistic phage combinations at MOIs of 10^0^ and 10^2^ (Fig. S7) and found that although powerful antibacterial effects were seen in the majority of these combinations even at lower MOIs, only 4 and 2 of the 6 combinations produced synergistic effects at MOIs of 10^2^ and 10^0^, respectively, suggesting that these synergistic effects work best at higher MOIs. These findings are perhaps unsurprising considering that most Bcc phages exhibit maximized antibacterial effects at higher MOIs (Fig. 1, Fig. S2 and S3), and suggest that polyphage therapy of Bcc infections may require high MOIs for synergistic antibacterial effects. Interestingly, both DC1 and KS14 have positive effects on the growth of B. cenocepacia AU41264 at an MOI of 10^0^ and this effect also translated to the combination of these phages, providing the only instance of a two-phage cocktail with a negative ${\mathrm{GRC}}_{x_{m}+y_{m}}$ and thus a positive effect on bacterial growth (Fig. S7I). Nevertheless, synergistic antibacterial effects were preserved at lower MOIs for many phage combinations (Fig. S7A to D, and G and H), suggesting that MOI may play a role in the synergistic interactions of some phages but not others and thereby implying that different mechanisms of phage-phage synergy may be at play. Taken together, these findings demonstrate that both high and low-*f*_(s.lys)_ LC phages can greatly improve the antibacterial effectiveness of phage cocktails, often through mathematically proven synergistic interactions, and thus reveal a novel therapeutic role for LC phages.

**TABLE 1:** Synergistic, additive, antagonistic, and autonomous growth reduction interactions between Bcc^*a*^ phages on susceptible strains of Burkholderia cenocepacia

Host	Phage pair	Condition								Interpretation
		Autonomous activity by phage *x*?			Autonomous activity by phage *y*?			Interaction		
		GRC(*x_m_* + *y_m_*) vs GRC(*x_m_*)			GRC(*x_m_* + *y_m_*) vs GRC(*y_m_*)			GRC(*x_m_* + *y_m_*) vs. GRC[(*x_m_*)(*y_m_*)]		
		Outcome	Sign	*P*	Outcome	Sign	*P*	Sign	*P*	
C6433	*x* = KS14	No	>	0.0026	Yes	=	0.9385^*b*^	<	0.0007	Autonomous activity
	*y* = KS5									
C6433	*x* = KS14	No	>	<0.0001	Yes	=	0.0692^*b*^	=	0.1739^*b*^	Autonomous activity
	*y* = AH2									
C6433	*x* = KS14	No	>	<0.0001	No	>	<0.0001	>	0.0008	Synergistic interaction
	*y* = KL1									
C6433	*x* = KS14	No	>	<0.0001	No	>	0.0057	>	0.0097	Synergistic interaction
	*y* = DC1									
C6433	*x* = KS5	No	>	<0.0001	Yes	=	0.3591^*b*^	=	0.2427^*b*^	Autonomous activity
	*y* = AH2									
C6433	*x* = KS5	No	>	<0.0001	No	>	0.0009	=	0.1307^*b*^	Additive activity
	*y* = KL1									
C6433	*x* = KS5	No	>	<0.0001	No	>	0.0078	>	0.0276	Synergistic interaction
	*y* = DC1									
C6433	*x* = AH2	Yes	=	0.0515^*b*^	No	>	<0.0001	<	0.0264	Autonomous activity
	*y* = KL1									
C6433	*x* = AH2	Yes	=	0.0923^*b*^	No	>	0.0078	<	0.0005	Autonomous activity
	*y* = DC1									
C6433	*x* = KL1	No	>	<0.0001	No	>	0.0089	=	0.5920^*b*^	Additive activity
	*y* = DC1									
K56-2	*x* = KS4M	Yes	=	0.5130^*b*^	No	<	0.0347	<	0.0182	Autonomous activity
	*y* = KS5									
K56-2	*x* = KS4M	No	>	0.0008	Yes	=	0.1321^*b*^	=	0.0583^*b*^	Autonomous activity
	*y* =JG068									
K56-2	*x* = KS4M	No	>	0.0030	Yes	=	0.0949^*b*^	<	0.0025	Autonomous activity
	*y* = KS9									
K56-2	*x* = KS5	Yes	=	0.1744^*b*^	No	>	0.0089	=	0.1061^*b*^	Autonomous activity
	*y* = JG068									
K56-2	*x* = KS5	Yes	=	0.1368^*b*^	No	>	0.0466	=	0.0925^*b*^	Autonomous activity
	*y* = KS9									
K56-2	*x* = JG068	No	>	0.0138	Yes	=	0.0876^*b*^	=	0.0608^*b*^	Autonomous activity
	*y* = KS9									
PC715J	*x* = KS14	Yes	=	0.0863^*b*^	Yes	=	0.2417^*b*^	<	0.0024	Autonomous activity
	*y* = KL1									
AU41264	*x* = KS14	No	>	0.0003	No	>	0.0157	=	0.2727^*b*^	Additive activity
	*y* = KL1									
AU41264	*x* = KS14	No	>	0.0006	No	>	<0.0001	>	0.0088	Synergistic interaction
	*y* = DC1									
AU41264	*x* = KL1	No	>	0.0143	No	>	<0.0001	=	0.0980^*b*^	Additive activity
	*y* = DC1									
AU41386	*x* = KS5	No	>	0.0131	No	>	0.0035	=	0.0774^*b*^	Additive activity
	*y* = AH2									
AU41386	*x* = KS5	No	>	0.0175	No	>	0.0070	=	0.6356^*b*^	Additive activity
	*y* = KL1									
AU41386	*x* = KS5	No	>	0.0104	No	>	0.0002	=	0.7014^*b*^	Additive activity
	*y* = DC1									
AU41386	*x* = KS5	No	>	0.0072	Yes	=	0.6379^*b*^	=	0.2716^*b*^	Autonomous activity
	*y* = JG068									
AU41386	*x* = KS5	Yes	=	0.1078^*b*^	No	>	0.0040	=	0.1206^*b*^	Autonomous activity
	*y* = KS9									
AU41386	*x* = AH2	Yes	=	0.3590^*b*^	No	>	<0.0001	<	<0.0001	Autonomous activity
	*y* = KL1									
AU41386	*x* = AH2	Yes	=	0.5442^*b*^	Yes	=	0.7920^*b*^	=	0.0926^*b*^	Autonomous activity
	*y* = DC1									
AU41386	*x* = AH2	No	>	0.0010	Yes	=	0.1039^*b*^	=	>0.9999^*b*^	Autonomous activity
	*y* = JG068									
AU41386	*x* = AH2	No	>	0.0066	No	>	<0.0001	>	0.0261	Synergistic interaction
	*y* = KS9									
AU41386	*x* = KL1	No	>	0.0001	No	<	0.0179	<	<0.0001	Antagonistic interaction
	*y* = DC1									
AU41386	*x* = KL1	No	>	0.0054	Yes	=	0.1542^*b*^	=	0.7879	Autonomous activity
	*y* = JG068									
AU41386	*x* = KL1	No	>	<0.0001	No	>	<0.0001	>	0.0063	Synergistic interaction
	*y* = KS9									
AU41386	*x* = DC1	No	>	<0.0001	Yes	=	0.4741^*b*^	=	0.6263^*b*^	Autonomous activity
	*y* = JG068									
AU41386	*x* = DC1	Yes	=	0.1452^*b*^	No	>	<0.0001	=	0.3185^*b*^	Autonomous activity
	*y* = KS9									
AU41386	*x* = JG068	No	<	0.0207	Yes	=	0.0728^*b*^	<	0.0240	Autonomous activity
	*y* = KS9									
PC184	*x* = DC1	No	>	0.0033	Yes	=	0.2289^*b*^	=	0.0519^*b*^	Autonomous activity
	*y* = JG068									

a Bcc, Burkholderia cepacia complex.

b Not significant.

## DISCUSSION

Although phage therapy is a promising potential solution to the global threat posed by antimicrobial resistance (AMR), the current paradigm of exclusively using obligately lytic phages severely restricts the range of organisms against which phage therapy can be utilized, simply due to the fact that OL phages appear to be rare for many pathogenic species, including members of the Bcc. In this study, we first address this issue from a theoretical perspective by proposing the more accurate term lysogenization-capable to describe phages which have the genetic capacity to form lysogens or are known to do so, at least to some extent, on at least one bacterial host, thereby emphasizing the fact that the mere capability to form stable lysogens is not the sole predictor of whether or not a phage will do so under therapeutically relevant conditions. This in turn implies that at least some LC phages may not form stable lysogens under such conditions and may therefore have therapeutic utility. We then proceed to examine this concept by introducing several novel metrics—Efficiency of Phage Activity, Growth Reduction Coefficient, and Stable Lysogenization Frequency—with which to quantify, in an *in vitro* setting, the potential therapeutic suitability of both LC and OL phages.

EPA, a modified form of the canonical EOP metric, gauges phage activity on solid medium by considering both plaques and non-plaque evidence of phage activity, including mottling and turbid clearance, which can be due to low-level productive infection but also nonproductive activity such as abortive infection (43) or lysis from without (44). Although these nonproductive activities may be therapeutically suboptimal, using the EPA allows quantitative characterization of the effects certain phages have on particular hosts through these mechanisms, as well as through productive infections, decreasing reliance on qualitative and thus subjective approaches such as the direct spot test (42, 62). EPA therefore serves as a preliminary screen by which to eliminate phage-host pairs for which very low (or no) activity is recorded and select more promising pairs which can then be investigated using more sophisticated approaches such as the GRC. The GRC, a modified form of the recently devised Virulence Index (22), measures the ability of a phage to reduce bacterial growth in a PKA across a therapeutically relevant period of time, rather than only during the logarithmic phase, thereby capturing the ability of the phage (or cocktail) to prevent outgrowths which can occur at later time points. In this study, we utilized an EPA threshold of ≥−5 to exclude phage-host pairs with very low activity, but subsequently found that even pairs for which EPA ≤ −4 are consistently associated with low GRCs (Fig. S4), suggesting that a more restrictive threshold might be advisable in future studies. Similarly, the utilized GRC threshold of GRC*_x_* or ${\mathrm{GRC}}_{x_{\max}}$ ≥ 0.05 was used because this study is exploratory and sought to investigate the relationships between as many phages, with even modest antibacterial effects, as possible. In future studies to select phages for use in model organisms or clinical trials, a much higher GRC threshold, perhaps GRC*_x_* or ${\mathrm{GRC}}_{x_{\max}}$ ≥ 0.50, might be advisable. Importantly, high EPA is not a good predictor of high GRC: while the EPA screening approach can be used to eliminate phage-host pairs with low activity, it cannot be used to suggest that high EPA pairs necessarily show therapeutic potential (Fig. S4). Substantial diversity in EPA, GRC, and MOI-GRC trends was noted among Bcc phages, both between distinct phages and within individual phages, depending on the specific host being targeted (Fig. 1 and 2; Fig. S1 and 2). These differences may be the result of differences in infection parameters such as phage-host affinity, infectivity, burst size, and time to lysis (63), but they could also be due to host factors such as receptor availability and phage resistance (8, 43). To further emphasize the importance of growth curve-based approaches such as the VI and GRC, we devised a derivative of the GRC termed endpoint GRC—which measures the growth reduction capacity of a phage using only the endpoint of bacterial growth—and found that this approach severely underestimates the GRC (Fig. 3), so endpoint approaches should only be used in circumstances where growth curve-based methods are not possible. *f*_(s.lys)_ is a novel variable which gauges the stable lysogen formation behaviors of LC phages, and the observed variation in this metric is crucial to our proposed paradigm of phages occupying points on a spectrum in terms of their tendency to form stable lysogens. Indeed, the *f*_(s.lys)_ of LC phages appears to vary widely with environmental factors such as temperature, infection medium, host, and potentially *in vivo* factors such as immune-mediated phage inactivation and the relative *in vivo* stability of the particle and prophage forms; it varies substantially between different LC phages as well (Fig. 4), which supports our conjecture that the genetic capacity to form lysogens is only a small part of the overall picture. Curiously, many integrating LC phages fail to produce any evidence of stable lysogen formation on particular hosts, possibly due to genetic incompatibilities between the *attP* and *attB* sites of the phage and host, respectively. Other phages interestingly exhibit low but non-zero *f*_(s.lys)_ on certain hosts; this could be caused by “leaky” integration sites, which lead to higher rates of spontaneous prophage induction, or perhaps by bacterial physiological responses which interfere with the establishment or maintenance of lysogeny in some strains (23, 25). The *f*_(s.lys)_ of the myovirus KS14 appears to be higher in nutrient-rich 1/2 LB and lower in M9 MM, possibly suggesting that lysogeny is favored under high-nutrient conditions in Bcc phages—a trend opposite to that described for the coliphage lambda (25)—but the overall effects of temperature and infection medium on *f*_(s.lys)_ are challenging to interpret and warrant further investigation.

Although the mechanistic underpinnings of the enormous variability in EPA, GRC, and *f*_(s.lys)_ reported among Bcc phages remain, for the most part, difficult to understand, the fact that such variability exists, particularly among LC phages, reiterates the fact that specific environmental and host conditions are key determinants of whether a phage behaves in a therapeutically suitable manner. Concordantly, the potential therapeutic utility of LC phages must therefore be determined on a case-by-case basis through rigorous evaluation of their GRC and *f*_(s.lys)_, rather than through the overly simplistic, black-and-white approach of disqualifying phages based on the presence of a lysogenic cassette. In comparing the GRC and *f*_(s.lys)_ of Bcc phages, we found a highly significant (R^2^ = 0.67; *P* < 0.0001) inverse relationship between these values for most phages which exhibited at least moderate (GRC ≥ 0.05) antibacterial activity (Fig. 5A), demonstrating that low-*f*_(s.lys)_ LC Bcc phages can have high GRC values and may therefore be therapeutically suitable. Taken together, these findings illustrate the flawed nature of the current paradigm of exclusively using OL phages by revealing that LC phages vary substantially in their therapeutic suitability, and that while high-*f*_(s.lys)_ LC phages are unsuitable on their own, low-*f*_(s.lys)_ LC phages can be as effective as OL phages in monophage therapy.

Although monophage applications may be useful in situations where only one phage is available for a particular host, the high risk of resistance development, and the desire to avoid the strategic mistakes which led to the current AMR catastrophe, have led most researchers, in both experimental and clinical settings, to instead use treatments combining multiple phages simultaneously (8, 29, 64–68). Recognizing the therapeutic importance of these polyphage cocktails, we used the GRC and *f*_(s.lys)_ metrics to explore the therapeutic usefulness of LC Bcc phages in equal-ratio two-phage cocktails; in particular, we sought to determine whether the effects of these two-phage cocktails result from significant, mathematically defined synergistic interactions. Although mathematically defined synergistic interactions have previously been reported for combinations of multiple antibiotics (39, 56–58) and combinations of single phages and antibiotics (59, 61), and several studies have demonstrated—without using rigorous mathematical definitions—improved antibacterial effects resulting from the combination of two or more phages (42, 69), to our knowledge, this is the first study to report mathematically defined synergistic and antagonistic interactions, as well as autonomous and additive effects, between two phages. Interestingly, we found that many of these two-phage combinations produced powerful antibacterial effects which were either equal to (additive) or greater than (synergistic) the multiplicative antibacterial effects of their constituents (Fig. 6; Fig. 7B and D; all data summarized in Table 1 and graphically shown in Fig. S6). Most importantly, neither low nor high-*f*_(s.lys)_ status in a constituent phage appears to impede the formation of cocktails with strong antibacterial activity because all of the phages participating in additive or synergistic combinations were LC, and high-*f*_(s.lys)_ LC phages were in fact present in 4 of the 6 synergistic combinations identified (Fig. 5B; Table 1), including KS14 and DC1 on B. cenocepacia C6433, which had the strongest recorded antibacterial effect (Fig. 7D). Furthermore, many of these combinations produce strong antibacterial effects even at lower MOIs (Fig. S7), meaning their synergistic effects could certainly be useful in therapeutic settings where lower MOIs are sometimes required. Taken together, these findings further suggest a shift from the current paradigm of exclusively using OL phages by revealing that LC phages may be rendered highly therapeutically useful through additive or synergistic interactions with other phages, which collectively produce powerful antibacterial effects. It is important to note, however, that this effect does not apply universally to all LC phages. On B. cenocepacia K56-2, for instance, KS4M (*f*_(s.lys)_ = 1) failed to substantially improve the effectiveness of any phage with which it was paired, and in fact appeared to reduce the antibacterial activity of KS5 (Fig. 6D). Similarly, KS14 (*f*_(s.lys)_ = 0.92) does not improve the effectiveness of KL1 on PC715J (Fig. 6F), suggesting that at least some LC phage pairings are therapeutically ineffective; therefore, a case-by-case evaluation of LC phage combinations on relevant hosts remains indispensable.

When discussing the effects of polyphage cocktails, it is crucial to stress that although synergistic interactions are arguably the most interesting because they suggest interplays between individual phages, additive effects—so long as they substantially reduce bacterial growth—are equally useful from a therapeutic perspective. On the other hand, autonomous effects, in which the more effective phage governs the overall antibacterial effect (Fig. 7A), suggest that use of the combination is not detrimental but provides no advantage relative to the more effective phage on its own. Conversely, antagonistic interactions (Fig. 7C) and autonomous effects in which the less effective phage governs the antibacterial interaction suggest that these combinations should be avoided because they produce antibacterial effects which are lesser than those achievable using the more effective phage alone.

Although a mathematical analysis of antibacterial effects is certainly crucial for determining whether the effects of a particular phage combination are autonomous, additive, synergistic, or antagonistic, it must be acknowledged that this approach does not facilitate a mechanistic understanding of how these effects occur. Although infection parameters such as phage-host affinity, infectivity, burst size, and time to lysis may play a role in how phages can interact (22, 42, 63), these parameters remain largely unknown for Bcc phages.

Furthermore, whether the constituent phages of a cocktail utilize the same receptor could be useful for understanding their interactions, but primary receptors are known for only 3 of the 8 Bcc phages examined in this study (Table S2), rendering the interactions between them challenging to comprehend. For instance, in this study we report the first mathematically defined example of antagonism between two phages, KL1 and DC1 (Fig. 7C), but the mechanism behind this interaction remains elusive because the receptors of both phages remain unknown. Similarly, the mechanisms whereby KS4M and KS9, despite being less effective than their counterpart phages, govern the antibacterial effects of their combinations with KS5 and the OL phage JG068, respectively (Fig. S6P; Fig. S6AD), are also challenging to understand. Since KS9 is known to bind the lipid A motif of lipopolysaccharide (LPS) while JG068 binds the more distal O-antigen (see Table S2), KS9-resistant mutants with a truncated lipid A would also lack the O-antigen and could therefore possess cross-resistance to JG068. This may imply that KS9 is the dominant phage in the pair since resistance to it causes resistance to both phages, while the reverse is not true, which could explain why the antibacterial effects of KS9 govern the antibacterial effect of the KS9 and JG068 pair (Fig. S6AD). In general, phage pairs containing phages which bind the same or similar receptors, such as different components of the LPS, are theoretically likely to exhibit autonomous activity governed by the phage which binds the more core-proximal element of the receptor, since resistance to this phage may cause cross-resistance to the other phage in the pair. Autonomous activity could also be explained by the related concept of superinfection exclusion (Sie [31]); however, the status of the Sie groups is unknown for the Bcc phages investigated in this study, making this explanation difficult to investigate at this point.

A related phenomenon which could explain the autonomous activity observed for certain phage pairs is superinfection immunity, in which the excess lytic repressor of an existing prophage blocks a secondary infection by a highly similar phage by binding to the operator sequences of the secondary phage (8, 23). In phage pairs containing phages with sufficient similarity in their operator sequences, the phage with a higher stable lysogenization frequency might dominate the interaction because it generates lysogens which are immune to superinfection by the other phage in the pair. This phenomenon could potentially explain why, for instance, the autonomous interaction between KS4M and KS5 is dominated by KS4M even though it is the less effective phage (Fig. S6P; see Table 1). Because KS4M has a high *f*_(s.lys)_, it is conceivable that superinfection immunity to KS5 by KS4M lysogens prevents the effectiveness of the former phage, possibly explaining the dominance of KS4M in this combination; however, this explanation is speculative because whether KS4M provides superinfection immunity against KS5 is unknown. The extent to which superinfection immunity causes autonomous activity is difficult to assess because the superinfection immunity profiles of Bcc phages remain uncharacterized, but it may play a lesser role than other factors since superinfection immunity occurs only between closely related phages.

The effects of similar receptors, superinfection exclusion, and superinfection immunity likely interact in phage pairs, along with the effects of infection parameters such as phage-host affinity, infectivity, burst size, and time to lysis, and the relative contributions of these various factors to the development of autonomous activity and possibly antagonistic interactions will be fascinating to uncover as more is learned about these parameters among Bcc phages.

Additive effects and synergistic interactions most likely result from combining phages which have particular infection parameters, as described previously, and the interplay between these parameters is likely most favorable in combinations of phages which interact synergistically (63). We suspect, however, that other factors may also be at least partially responsible for the synergistic interactions observed in this study. First, high-*f*_(s.lys)_ phages were present in 4 of these 6 combinations (see Table 1), raising the possibility that the formation of lysogens which can be targeted by the other phage in the cocktail without superinfection immunity could potentially play a role in the observed synergistic effect. Second, the other two combinations for which synergistic interactions were observed included phages which have distinct primary receptors (Table 1; Table S2), raising the possibility of synergy due to the low probability of simultaneous development of resistance to both phages (65). While tantalizing, these conjectures currently remain speculative, and improved understanding of Bcc phage receptors, infection parameters, and the role of Bcc lysogens is needed to shed light on the mechanisms behind these interactions.

In this study, we explored the effectiveness of two-phage combinations only at a ratio of 1:1, but our results suggest that GRC appears to be maximized at lower MOIs in a small minority of Bcc phage-host pairs (Fig. 1; Fig. S2), suggesting that combinations in which one phage is present in trace amounts relative to the other might produce even stronger antibacterial effects in certain two-phage combinations. This idea was briefly explored by Storms et al. (22), who found that 1:1- and 1:3-ratio combinations of the coliphages T7 and T5 had distinct VI values, although neither combination was significantly different than monophage treatment with T7. This suggests that future work in optimizing these polyphage combinations might focus on exploring the effects of ratios, using the MOI-GRC trends identified in this study as a baseline for determining the MOIs at which the GRCs of individual Bcc phages are optimized.

Because our primary objective was to assess the potential therapeutic suitability of LC Bcc phages, both in monophage and polyphage applications, based on *in vitro* evaluation using our newly developed parameters, we did not focus on the effectiveness of these phages *in vivo*. Although *in vivo* polyphage treatment trials for Bcc infections have never been published, treatment of Bcc infections in G. mellonella larvae using combinations of antibiotics and single phages demonstrated antibacterial effects comparable to those seen in *in vitro* phage-antibiotic anti-Bcc PKAs, suggesting that polyphage treatments might have similar antibacterial effects *in vivo* to those *in vitro* (30). Whether the synergistic interactions reported here might be maintained *in vivo* is difficult to predict, however, and warrants further investigation. Intriguingly, KS14 appears to have a decreased *f*_(s.lys)_ in the hemolymph of G. mellonella (Fig. 4A), meaning that if the synergistic interactions identified in several of our combinations are indeed at least partially reliant on lysogen formation, the magnitude of the synergistic effect may be diminished *in vivo*. Conversely, synergistic interactions based on receptor identity would likely be preserved *in vivo*, assuming that phage infectivity is unaffected under such conditions. Regardless, *in vivo* polyphage treatments of Bcc infections are the next logical step in determining whether these phages may be used in human trials and should be explored at length in subsequent studies.

Finally, efforts should be made to study the effects of phage-antibiotic cocktails containing high-*f*_(s.lys)_ LC phages and compounds which use activation of the cellular stress response to induce these phages into their lytic cycles. In a recent seminal study, Al-Anany et al. (31) convincingly demonstrated that the LC coliphage HK97 can be therapeutically effective when paired with ciprofloxacin, since this compound triggers induction of the HK97 prophage to the lytic cycle, thereby improving overall bacterial clearance. Although we are unaware of any studies investigating combined treatments with LC phages and complement, this essential component of the innate immune system also stresses bacterial cells and could therefore improve overall growth reduction by triggering the induction of high-*f*_(s.lys)_ LC prophages. Crucially, the *f*_(s.lys)_ metric introduced in this study can be used to quantify the tendency of phages to form stable lysogens and can thereby serve as an excellent screen for identifying phages that are likely to synergize with prophage-inducing compounds.

In conclusion, it is reasonable to expect that most existing phages may be genetically capable of forming lysogens due to the evolutionary benefits of lysogenization, meaning that the existing paradigm of exclusively utilizing obligately lytic phages in phage therapy is therefore impractical. Viewed broadly, lysogenization among phages can pose a severe problem to the application of phage therapy, but this problem can be overcome if lysogenization-capable phages are used selectively and strategically. To that end, all LC phages must be evaluated for their tendency to form stable lysogens and ability to reduce bacterial growth, using the *f*_(s.lys)_ and GRC metrics, respectively, as part of their initial characterization. Low-*f*_(s.lys)_–high-GRC phages may be used on their own if no alternatives are available, while both low- and high-*f*_(s.lys)_ phages may be used in polyphage cocktails in which antibacterial activity is maximized through additive or synergistic interactions. These approaches can be increasingly elaborated by combining these phages with specific stress-inducing antibiotics, with the ultimate aim of constructing an LC polyphage-antibiotic cocktail which can completely abolish bacterial growth.

## MATERIALS AND METHODS

### Organisms and media.

Seventeen strains belonging to the Bcc were examined as part of this study, including 9 B. cenocepacia, 2 B. ambifaria, 2 B. multivorans, 1 B. stabilis, and 3 B. dolosa. Fifteen of these strains were clinical isolates from CF patients in several countries, including Canada (*n* = 6), the United States (*n* = 5), Australia (*n* = 2), the United Kingdom (*n* = 1), and Belgium (*n* = 1), while two strains were environmental isolates from rhizosphere samples (Table S1). Virtually all experiments in this study were conducted using half-strength Luria-Bertani broth (1/2 LB [70]; 5g/L bacto tryptone, 2.5g/L yeast extract, 2.5g/L NaCl) or 1/2 LB 1.5% (wt/vol) agar plates, while 20% glycerol full-strength LB was used for long-term storage of bacterial strains at −80°C. Bacteria were incubated statically on 1/2 LB 1.5% (wt/vol) agar plates at 30°C for 48 h or until colonies appeared, and single colonies were cultured in 5 mL 1/2 LB broth at 30°C and at 225 rpm in a gyratory shaker for precisely 18 h. After 18 h, bacterial cultures were plated for viable CFU and their OD_600_ was measured using a Nanodrop spectrophotometer. OD_600_-CFU equivalencies for this time point were then constructed for each bacterial strain and used to approximate viable CFU in 18-h cultures. Bacterial cultures were serially diluted in fresh 1/2 LB broth to a starting inoculum of 10^5^ or 10^3^ CFU (see Table S1), which roughly corresponds to the 50% lethal dose (LD_50_) of most Bcc strains in G. mellonella (greater wax moth) larvae, the organism in which Bcc PT is most frequently studied (71).

Eight Bcc phages previously isolated and characterized by members of the Dennis Laboratory (8, 12, 36, 72–74), including seven experimentally LC phages, AH2, DC1, KL1, KS4M, KS5, KS9, and KS14, as well as the OL phage JG068, which serves as a comparator, were examined in this study (Table S2). All phages were stored at 4°C in suspension medium (SM [75]; 5.8g/L NaCl, 2g/L MgSO_4_, 50 mL/L of 1 M Tris-HCl) until required, and maintenance of high phage titer was routinely verified. Phage amplification was conducted using the double-layer agar approach (30), and lysate containing amplified phage was resuspended in SM, scraped off plates and centrifuged at 12,000 rpm for 7 min, and the supernatant was sterilized using a 0.45-μm MCE filter (Merck Millipore, Ireland). Filter-sterilized lysate was subsequently titered using the double-layer agar approach (30), and these techniques were performed repeatedly until sufficiently high phage titers (10^9^ to 10^10^ PFU/mL) were obtained. Phages DC1 and KS14 were amplified only on B. cenocepacia C6433 and phages KS9 and JG068 only on B. cenocepacia K56-2, while phages AH2, KL1, KS4M, and KS5 were amplified on both strains, giving rise to variants with slightly altered host ranges (not shown); however, only the more effective (on relevant strains) variants of these phages were used in this study.

### Efficiency of Phage Activity assay.

This method is procedurally similar to the previously described Efficiency of Plating Assay (40) in which a confluent lawn of the target bacterium is poured onto a 1/2 LB 1.5% (wt/vol) agar plate using the double-agar method described previously (30). Phages are serially diluted in SM in order-of-magnitude steps from 10^10^ to 10^3^ PFU/mL, and 0.005 mL of each concentration is then spotted in triplicate onto the bacterial lawn, meaning the lowest concentration at which a spot would contain at least one phage is 10^3^ PFU/mL; this, therefore, is the lowest concentration at which evidence of phage activity is theoretically possible. Plates are incubated statically at 30°C for 18h and are then inspected visually. The lowest concentration at which evidence of phage activity (plaques, mottling, turbid clearance) is visible is then compared to the lowest concentration at which such evidence is theoretically possible, using equation 1 to obtain the EPA score. EPA scores are unitless and range from 0 to −7, where EPA < −7 indicates no detectable sensitivity to phage activity.

### Planktonic Killing Assay.

All experiments were conducted using flat-bottomed 24-well plates (Corning, USA). Here, 0.1 mL of a 10^6^ CFU/mL diluted culture was added to each relevant well and mixed with 0.1 mL phage stock at order-of-magnitude concentrations ranging from 10^5^ to 10^10^ PFU/mL, thereby generating an MOI range of 10^−1^ to 10^4^. Slightly different values were used for B. cenocepacia K56-2 because the LD_50_ of this strain in G. mellonella is lower (see Table S1), but the same MOI range was maintained. Assays with two-phage combinations were performed using the same protocol, but only at the maximum available MOI, either 10^3^ or 10^4^. Bacteria and phage were permitted to mix for 10 min at room temperature and subsequently covered with 2 mL fresh 1/2 LB broth. Positive-control wells contained 0.1 mL of bacteria mixed with 0.1 mL SM, covered with 2 mL 1/2 LB, while negative-control wells received only 0.1 mL SM and were covered with 2.1 mL 1/2 LB to maintain an identical volume. Plates were incubated in either a BioTek Epoch 2 microplate spectrophotometer or a BioTek Cytation 5 Multi-Mode Reader at 30°C with continuous shaking at 237 rpm for 48 h, and OD_600_ readings were collected every hour. We used a 48-h growth period for all strains because this is the endpoint time typically used *in vivo* experiments using G. mellonella, the organism in which Bcc PT is most frequently studied (71). At least three biological replicates were conducted at each MOI for each phage and two-phage combination, and data from biological replicates were then averaged to produce a single growth curve at each MOI. Generated growth curves were analyzed using the Growth Reduction Coefficient approach, which uses the area under the growth curve to provide an estimate of the ability of the phage (or cocktail) to reduce bacterial growth (see Results).

### Stable lysogenization frequency assays.

*In vitro f*_(s.lys)_ experiments were performed using flat-bottomed 24-well plates (Corning, USA), with the same bacterial inoculum, phage concentration, and MOI ranges, and general procedures as described for PKAs, but four biological replicates were conducted for each phage-host pair and plates were incubated at either 30°C or 37°C at 225 rpm in a gyratory shaker for precisely 48 h. After 48 h, 1 mL of surviving cells was centrifuged for 5 min at 12 000 rpm to remove phage and washed with 0.5 mL of fresh 1/2 LB. This procedure was repeated three times to remove phage contamination and surviving cells were plated for single colony isolation. After 48 h of static growth at 30°C, 15 colonies were randomly selected from each of the four plates (biological replicates), for a total of 60 colonies per phage. Colonies were PCR-screened for lysogeny status using phage-specific primers and average *f*_(s.lys)_ values for the four replicates were calculated using equation 9 (see Results). *In vitro* assays with KS14 were conducted in M9 minimal medium (M9 MM [76]; 200 mL/L 5× M9 Salts, 2g/L MgSO_4_, 0.1g/L CaCl_2_, 4g/L glucose) and artificial cystic fibrosis sputum medium (ACFSM [77]), in addition to 1/2 LB, while assays with all other phages were conducted only in 1/2 LB. *In vivo f*_(s.lys)_ experiments were performed only with phage KS14 in the hemolymph of G. mellonella (greater wax moth) larvae. For each KS14-host pair, 12 larvae (3 technical replicates × 4 biological replicates) were injected sequentially with 10^5^ CFU and 10^8^ PFU of KS14 (MOI = 10^3^), with 2-h intervals between injections, as previously described (71), and larvae were incubated at 30°C and 37°C in the dark for 48 h after injection. After 48 h, larvae were euthanized via decapitation and hemolymph was extracted. The hemolymph of technical replicate larvae was pooled to form four biological replicate stocks, from which survivor colonies were isolated and PCR-screened for lysogeny status as described above.

### Statistical analysis.

In all figures and tables, data points for GRC and *f*_(s.lys)_ represent the mean values of at least three and four biological replicates, respectively. Error bars represent standard error of the mean (SEM) of all biological replicates, and errors reported for values used to statistically compare ${\mathrm{GRC}}_{x_{m}}$, ${\mathrm{GRC}}_{x_{m}+y_{m}}$ and ${\mathrm{GRC}}_{\left[(x_{m})(y_{m})\right]}$ values (see equations 10 to 14 in Results) are the summations of errors reported for the components of these values. GraphPad Prism version 9.3.1 (San Diego, CA, USA) was used to compute the areas under all bacterial growth curves using the trapezoid approximation method, as well as to conduct all statistical analyses, including linear regression between variables and Student’s *t* tests (with Welch’s correction for unequal variances) for comparisons between the GRCs of single phages and two-phage cocktails. *P* < 0.05 was considered statistically significant.
