# Supplementary material for: Synergistic Interactions among Burkholderia cepacia Complex-Targeting Phages Reveal a Novel Therapeutic Role for Lysogenization-Capable Phages
Source: Microbiol Spectr. 2023 May 17;11(3):e04430-22. doi: 10.1128/spectrum.04430-22 (PMC10269493; doi:10.1128/spectrum.04430-22)
Supplement: Supplemental file 1 — Supplemental material. Download spectrum.04430-22-s0001.pdf, PDF file, 9.4 MB [file spectrum.04430-22-s0001.pdf]

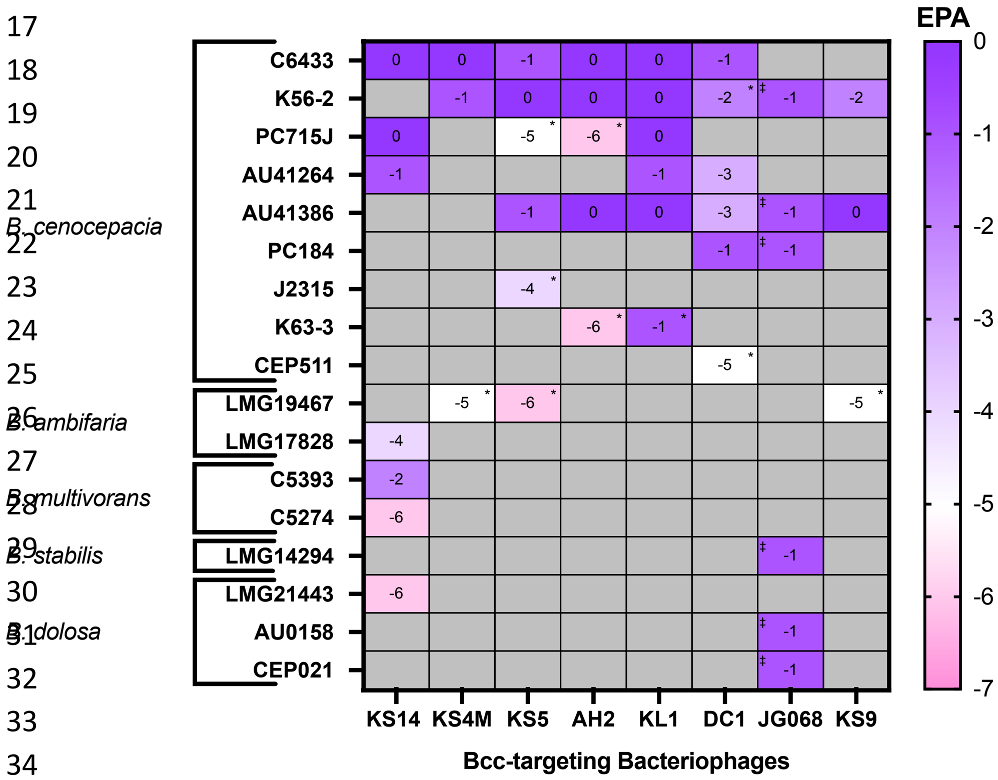

Supplementary Figure 1: Efficiency of Phage Activity (EPA) of Bcc-targeting phages on susceptible Bcc species. Solid medium infections were conducted using a soft agar overlay serial dilution spotting assay, and EPA values were calculated for all phage-host pairs using **equation 1**. EPA values range from a maximum of 0 to a minimum of -7, while EPA < -7 indicates no phage sensitivity and is indicated by cells shaded in grey. Cells designated with * indicate phage-host pairs in which phages produce zones of bacterial lawn weakening or extremely turbid clearing, rather than discrete, readily countable plaques. Cells designated with ‡ indicate phage-host combinations containing the OL phage JG068. An arbitrary threshold of EPA ≥ -5 was used to select phage-host pairs for further investigation, while phage-host pairs with EPA < -5 were excluded from further research.

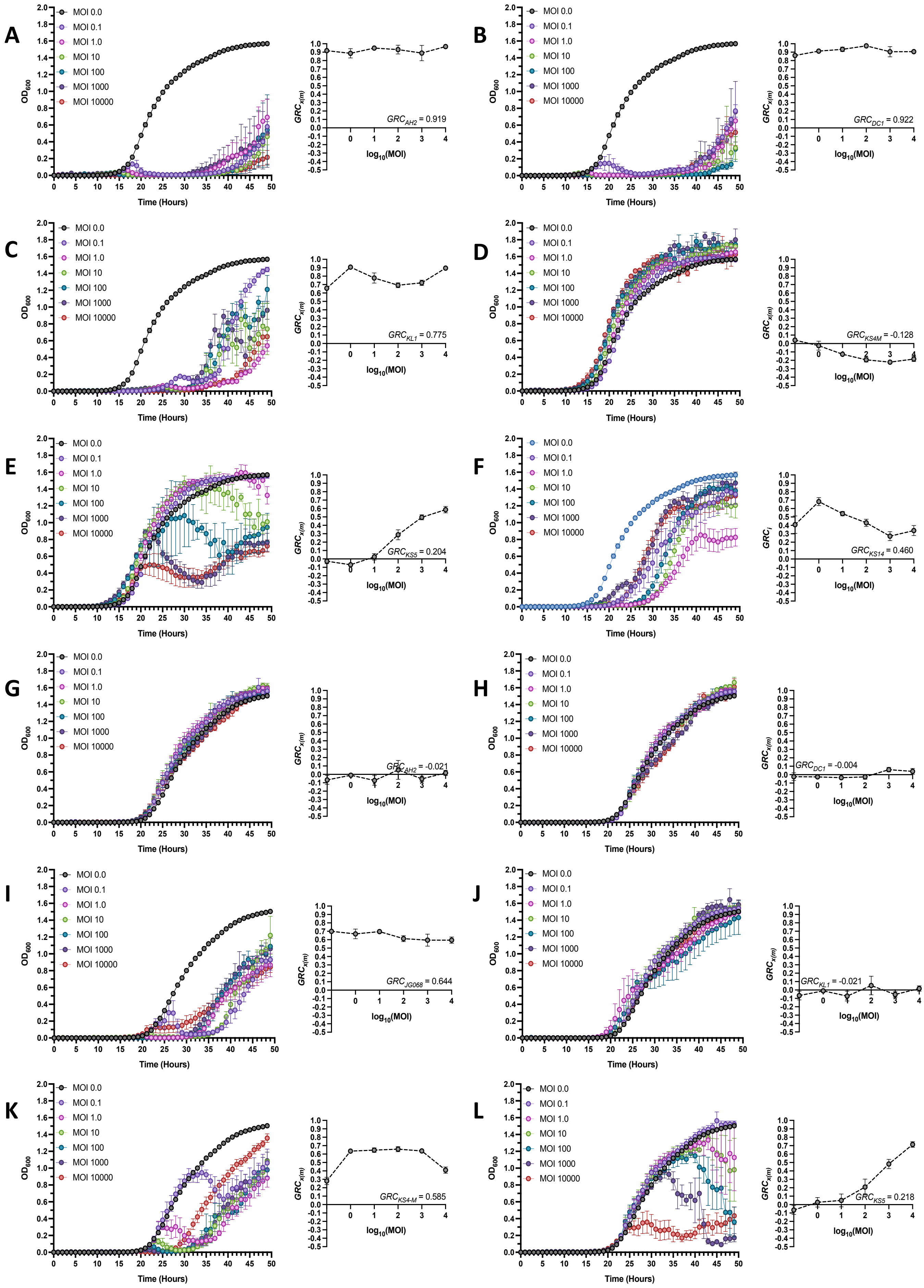
A
B
C
D
E
F
G
H
I
J
K
L
MOI 0.0
MOI 0.1
MOI 1.0
MOI 10
MOI 100
MOI 1000
MOI 10000
OD600
Time (Hours)
GRCx(m)
GRCi
log10(MOI)
GRCAH2 = 0.919
GRCDC1 = 0.922
GRCKL1 = 0.775
GRCKS4M = -0.128
GRCKS5 = 0.204
GRCKS14 = 0.460
GRCAH2 = -0.021
GRCDC1 = -0.004
GRCJG068 = 0.644
GRCKL1 = -0.021
GRCKS4-M = 0.585
GRCKS5 = 0.218

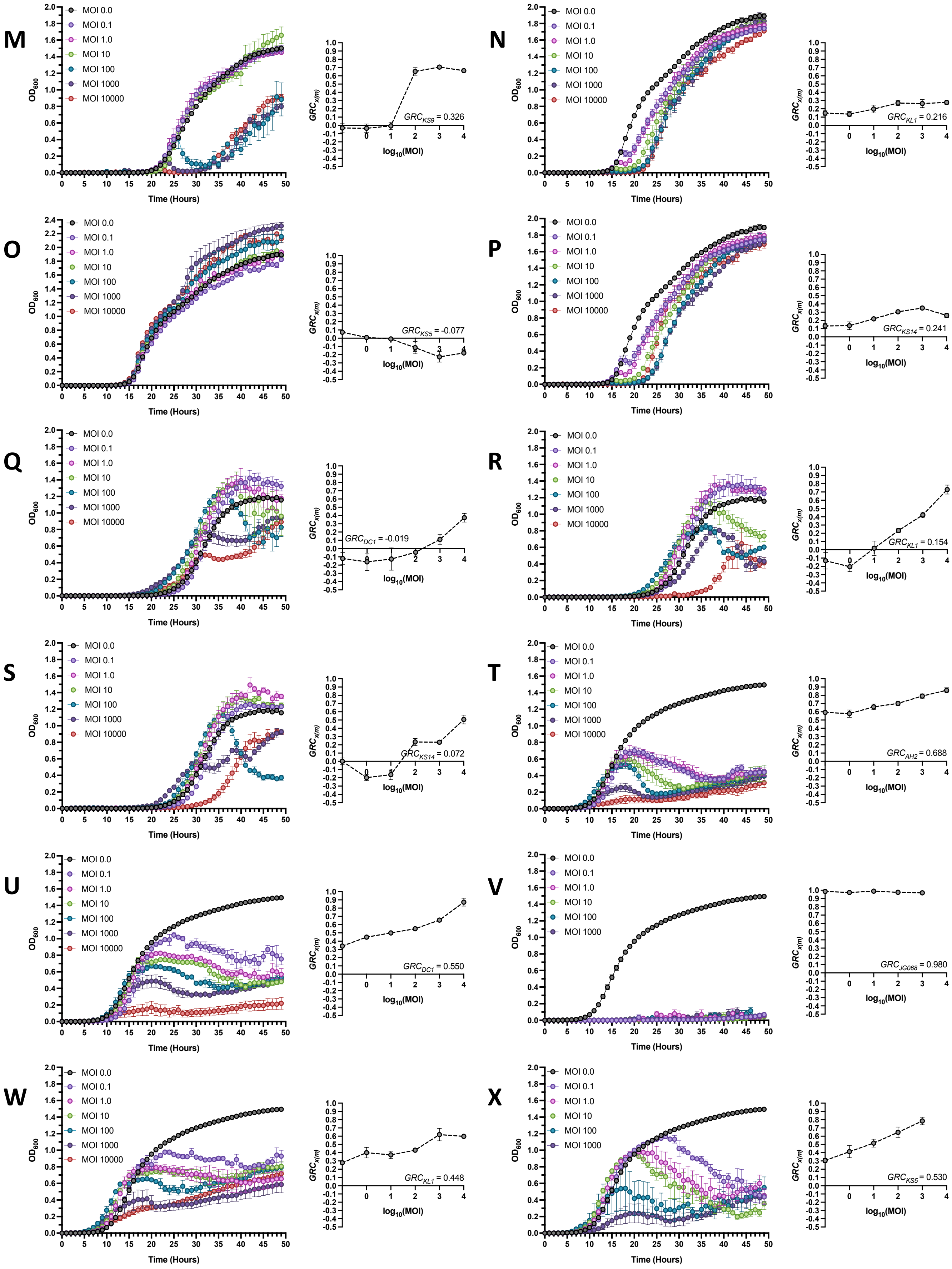
M
MOI 0.0
MOI 0.1
MOI 1.0
MOI 10
MOI 100
MOI 1000
MOI 10000
OD600
Time (Hours)
$GRC_{x(m)}$
GRC_{KS9} = 0.326
log_{10}(MOI)
N
MOI 0.0
MOI 0.1
MOI 1.0
MOI 10
MOI 100
MOI 1000
MOI 10000
OD600
Time (Hours)
$GRC_{x(m)}$
GRC_{KL1} = 0.216
log_{10}(MOI)
O
MOI 0.0
MOI 0.1
MOI 1.0
MOI 10
MOI 100
MOI 1000
MOI 10000
OD600
Time (Hours)
$GRC_{x(m)}$
GRC_{KS5} = -0.077
log_{10}(MOI)
P
MOI 0.0
MOI 0.1
MOI 1.0
MOI 10
MOI 100
MOI 1000
MOI 10000
OD600
Time (Hours)
$GRC_{x(m)}$
GRC_{KS14} = 0.241
log_{10}(MOI)
Q
MOI 0.0
MOI 0.1
MOI 1.0
MOI 10
MOI 100
MOI 1000
MOI 10000
OD600
Time (Hours)
$GRC_{x(m)}$
GRC_{DC1} = -0.019
log_{10}(MOI)
R
MOI 0.0
MOI 0.1
MOI 1.0
MOI 10
MOI 100
MOI 1000
MOI 10000
OD600
Time (Hours)
$GRC_{x(m)}$
GRC_{KL1} = 0.154
log_{10}(MOI)
S
MOI 0.0
MOI 0.1
MOI 1.0
MOI 10
MOI 100
MOI 1000
MOI 10000
OD600
Time (Hours)
$GRC_{x(m)}$
GRC_{KS14} = 0.072
log_{10}(MOI)
T
MOI 0.0
MOI 0.1
MOI 1.0
MOI 10
MOI 100
MOI 1000
MOI 10000
OD600
Time (Hours)
$GRC_{x(m)}$
GRC_{AH2} = 0.688
log_{10}(MOI)
U
MOI 0.0
MOI 0.1
MOI 1.0
MOI 10
MOI 100
MOI 1000
MOI 10000
OD600
Time (Hours)
$GRC_{x(m)}$
GRC_{DC1} = 0.550
log_{10}(MOI)
V
MOI 0.0
MOI 0.1
MOI 1.0
MOI 10
MOI 100
MOI 1000
OD600
Time (Hours)
$GRC_{x(m)}$
GRC_{JG068} = 0.980
log_{10}(MOI)
W
MOI 0.0
MOI 0.1
MOI 1.0
MOI 10
MOI 100
MOI 1000
MOI 10000
OD600
Time (Hours)
$GRC_{x(m)}$
GRC_{KL1} = 0.448
log_{10}(MOI)
X
MOI 0.0
MOI 0.1
MOI 1.0
MOI 10
MOI 100
MOI 1000
OD600
Time (Hours)
$GRC_{x(m)}$
GRC_{KS5} = 0.530
log_{10}(MOI)

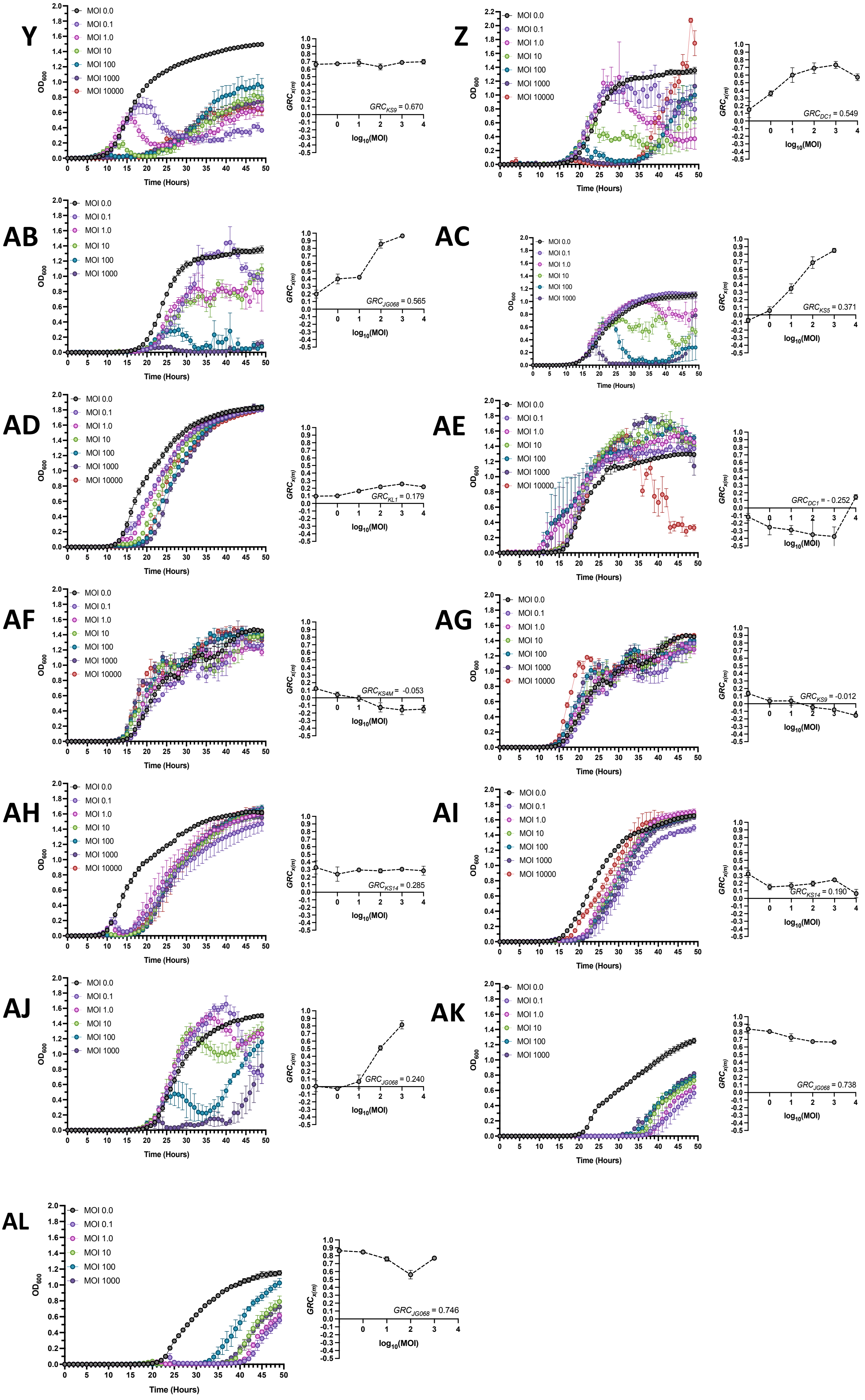
Y
OD600
Time (Hours)
MOI 0.0
MOI 0.1
MOI 1.0
MOI 10
MOI 100
MOI 1000
MOI 10000
GRCx(m)
log10(MOI)
GRC KS9 = 0.670
Z
GRC DC1 = 0.549
AB
GRC JG068 = 0.565
AC
GRC KS5 = 0.371
AD
GRC KL1 = 0.179
AE
GRC DC1 = - 0.252
AF
GRC KS4M = -0.053
AG
GRC KS9 = -0.012
AH
GRC KS14 = 0.285
AI
GRC KS14 = 0.190
AJ
GRC JG068 = 0.240
AK
GRC JG068 = 0.738
AL
GRC JG068 = 0.746

Supplementary Figure 2: Growth Reduction Trends of Phages Targeting the Bcc. Bacterial growth curves (left) and associated Growth Reduction Coefficient (GRC) curves (right) of Bcc phages on *Burkholderia cenocepacia* strains **C6433** [phages AH2(c) (**A**), DC1 (**B**), KL1(c) (**C**), KS4M(c) (**D**), KS5(c) (**E**), KS14 (**F**)], **K56-2** [phages AH2(k) (**G**), DC1 (**H**), JG068 (**I**), KL1(k) (**J**), KS4M(k) (**K**), KS5(k) (**L**), KS9 (**M**)], **PC715J** [phages KL1(c) (**N**), KS5(c) (**O**), KS14 (**P**)], **AU41264** [phages DC1 (**Q**), KL1(c) (**R**), KS14 (**S**)], **AU41386** [phages AH2(c) (**T**), DC1 (**U**), JG068 (**V**), KL1(c) (**W**), KS5(k) (**X**), KS9 (**Y**)], **PC184** [phages DC1 (**Z**), JG068 (**AB**)], **J2315** [phage KS5(k) (**AC**)], **K63-3** [phage KL1(c) (**AD**)], and **CEP511** [phage DC1 (**AE**)]; *Burkholderia ambifaria* strains **LMG 19467** [phages KS4M(k) (**AF**), KS9 (**AG**)], and **LMG 17828** [phage KS14 (**AH**)]; *Burkholderia multivorans* strain **C5393** [phage KS14 (**AI**); *Burkholderia stabilis* strain **LMG 14294** [phage JG068 (**AJ**)]; and *Burkholderia dolosa* strains **AU0158** [phage JG068 (**AK**)], and **CEP021** [phage JG068 (**AL**)]. All experiments were performed at standard conditions across the standard MOI range of 0.1 to 10000, except for those phage-host pairs depicted in graphs **V**, **X**, **AB**, **AC**, **AJ**, **AK**, **AL**, for which the maximum available MOI was 1000. Black lines represent bacterial growth without phage (MOI 0.0), while coloured lines represent each of the investigated MOIs. GRC_{x_m} values were computed for each MOI using **equation 3**, and the GRC_x values for all phage-host pairs were then calculated using **equation 6** and are presented here for each pair. Note that the y-axes in graphs **O** and **Z** are slightly longer due to unusually high cell density resulting from phage treatment. Bars represent standard error of the mean of at least three biological replicates.

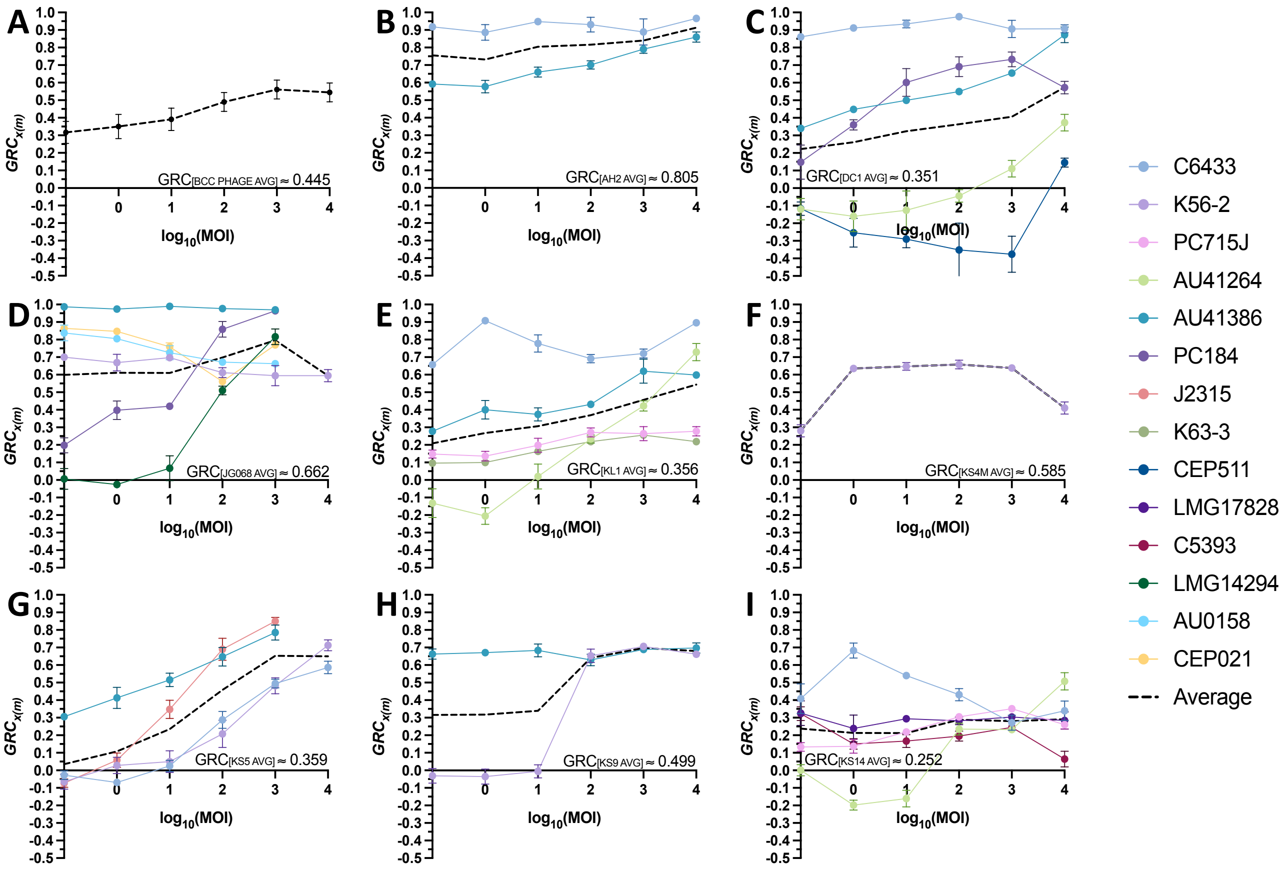

Supplementary Figure 3: Mean GRC_{x_m} vs MOI trends of Bcc-targeting phages. Mean GRC_{x_m} vs MOI trends and GRC_x values for a composite of all Bcc phages (**A**), as well as individual Bcc phages AH2 (**B**), DC1 (**C**), JG068 (**D**), KL1 (**E**), KS4M (**F**), KS5 (**G**), KS9 (**H**), and KS14 (**I**) on all tested host strains. For graphs depicting trends of individual phages (**B-I**), trends for each individual host strain are also shown to demonstrate the diversity of GRC_{x_m} vs MOI trends even for individual phages. Bars represent standard error of the mean for at least three biological replicates.

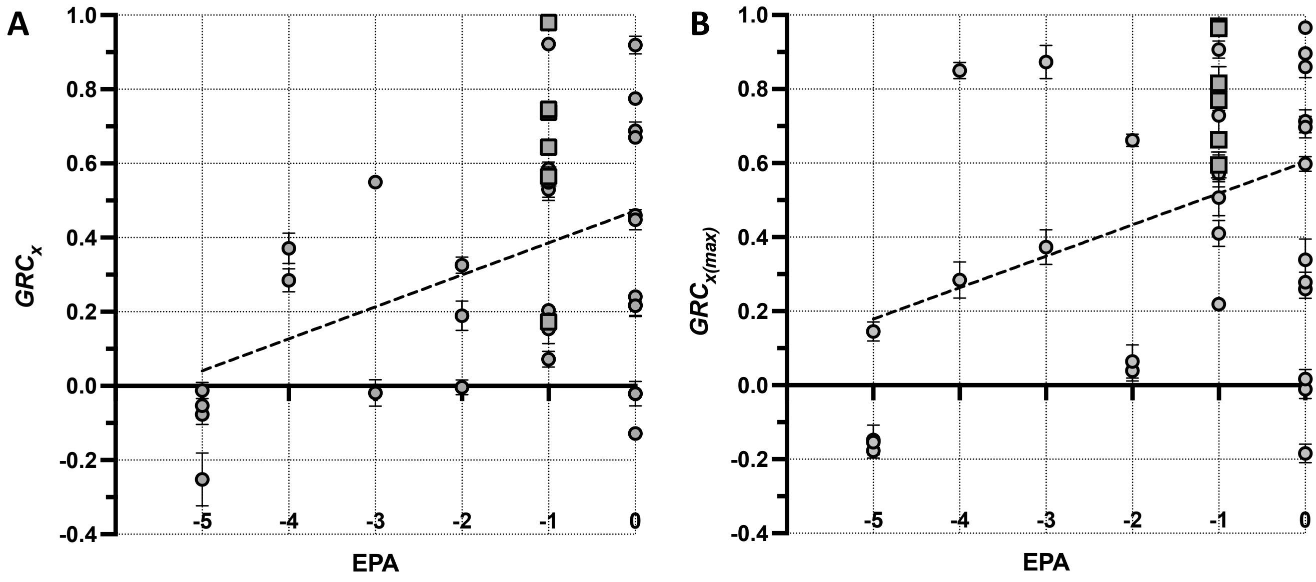

Supplementary Figure 4: Correlation between Efficiency of Phage Activity (EPA) and the Growth Reduction Coefficient (GRC) of Phages Targeting the Bcc. EPA, GRC_x, and $GRC_{x_{max}}$ values of all investigated Bcc phage-host pairs, for infections conducted at standard conditions across the standard MOI range of 0.1 to 10000, were calculated using **equations 1**, **6**, and **3**, respectively, and the EPA values were plotted against GRC_x (**A**) and $GRC_{x_{max}}$ (**B**) values using x-y scatterplots. Circles and squares represent phage-host pairs containing LC and OL phages, respectively. Dashed black lines represent lines-of-best-fit. Bars represent standard error of the mean for at least three biological replicates of GRC experiments.

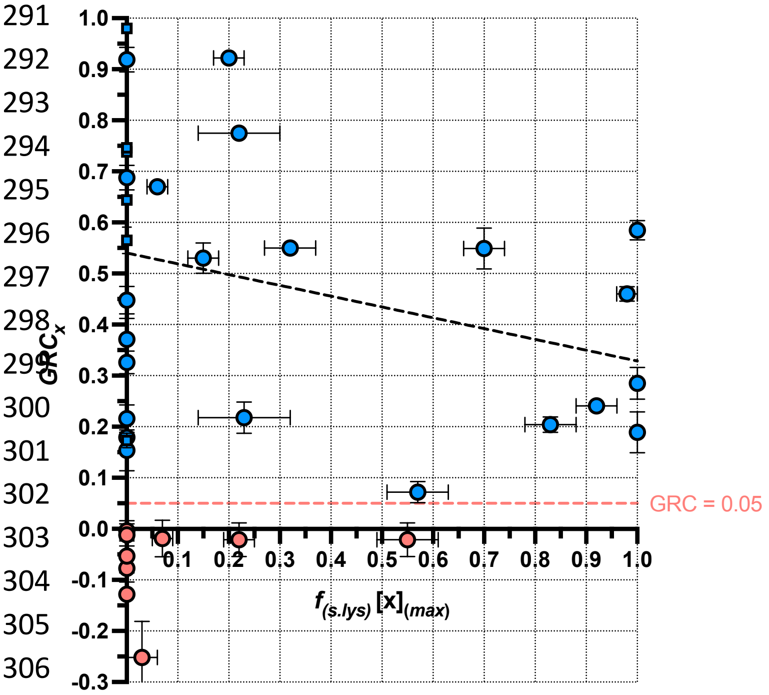

Supplementary Figure 5: Lack of Significant Correlation between GRC_x and $f_{(lys)}$ [x]$_{(max)}$. $f_{(lys)}$ values for infections conducted at the maximum available MOI, along with GRC_x values for infections conducted across the standard MOI range of 10^{-1} – 10^{4}, all at standard conditions, were calculated for each investigated Bcc phage-host pair using **equations 9** and **6**, respectively, and were plotted using an x-y scatterplot. Points shown in red represent phage-host pairs which did not satisfy the GRC ≥ 0.05 criterion and thus fall below the red dashed line (representing a GRC of precisely 0.05), while points shown in blue represent phage-host pairs which did satisfy the GRC ≥ 0.05 criterion. Circles and squares represent phage-host pairs containing LC and OL phages, respectively. The black dashed line represents the line-of-best-fit for points shown in blue. Vertical and horizontal error bars represent standard error of the mean of at least three and four biological replicates, respectively.

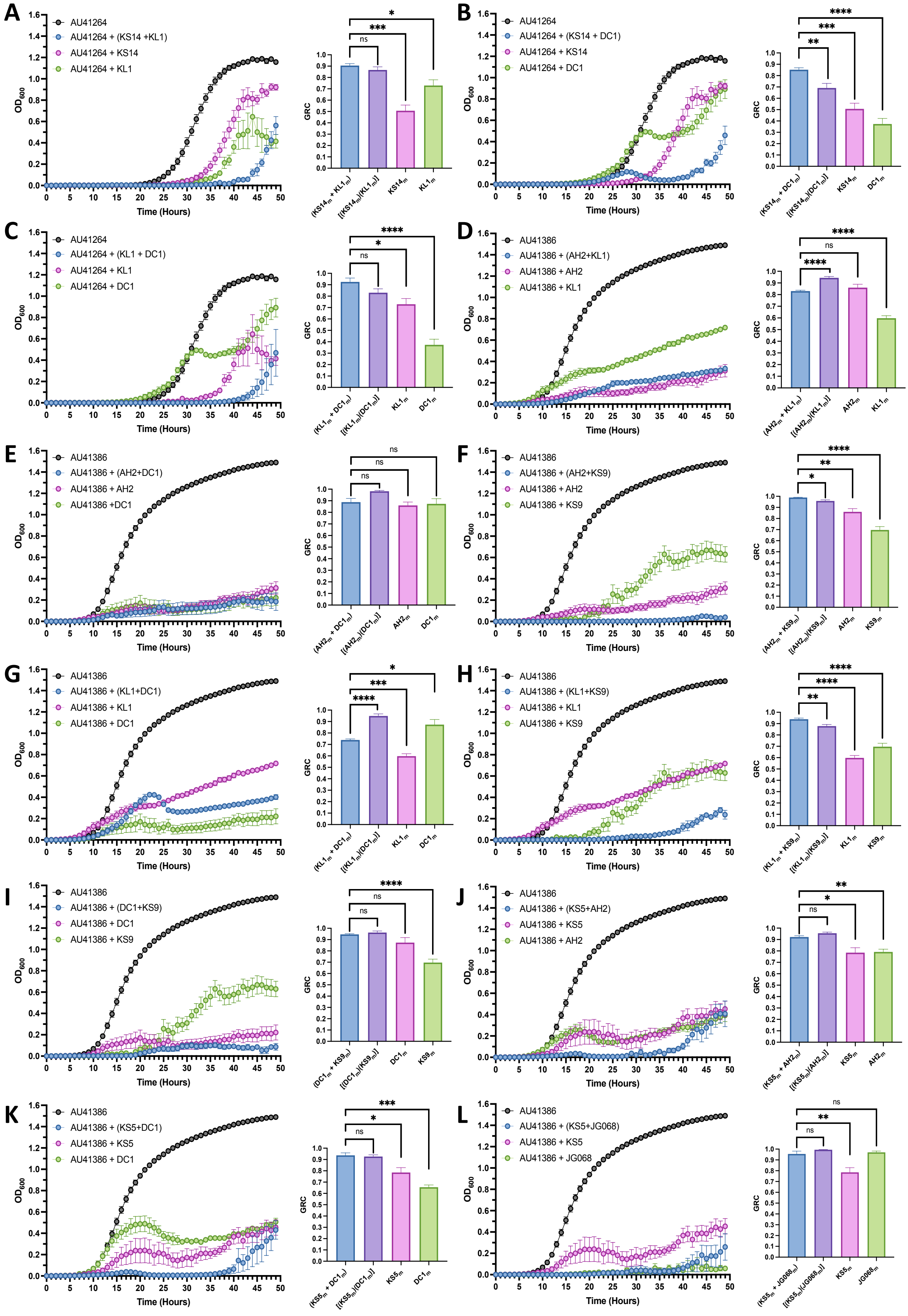
A
AU41264
AU41264 + (KS14 +KL1)
AU41264 + KS14
AU41264 + KL1
B
AU41264
AU41264 + (KS14 + DC1)
AU41264 + KS14
AU41264 + DC1
C
AU41264
AU41264 + (KL1 + DC1)
AU41264 + KL1
AU41264 + DC1
D
AU41386
AU41386 + (AH2+KL1)
AU41386 + AH2
AU41386 + KL1
E
AU41386
AU41386 + (AH2+DC1)
AU41386 + AH2
AU41386 +DC1
F
AU41386
AU41386 + (AH2+KS9)
AU41386 + AH2
AU41386 + KS9
G
AU41386
AU41386 + (KL1+DC1)
AU41386 + KL1
AU41386 + DC1
H
AU41386
AU41386 + (KL1+KS9)
AU41386 + KL1
AU41386 + KS9
I
AU41386
AU41386 + (DC1+KS9)
AU41386 + DC1
AU41386 + KS9
J
AU41386
AU41386 + (KS5+AH2)
AU41386 + KS5
AU41386 + AH2
K
AU41386
AU41386 + (KS5+DC1)
AU41386 + KS5
AU41386 + DC1
L
AU41386
AU41386 + (KS5+JG068)
AU41386 + KS5
AU41386 + JG068
OD600
Time (Hours)
GRC
ns
*
**
***
****

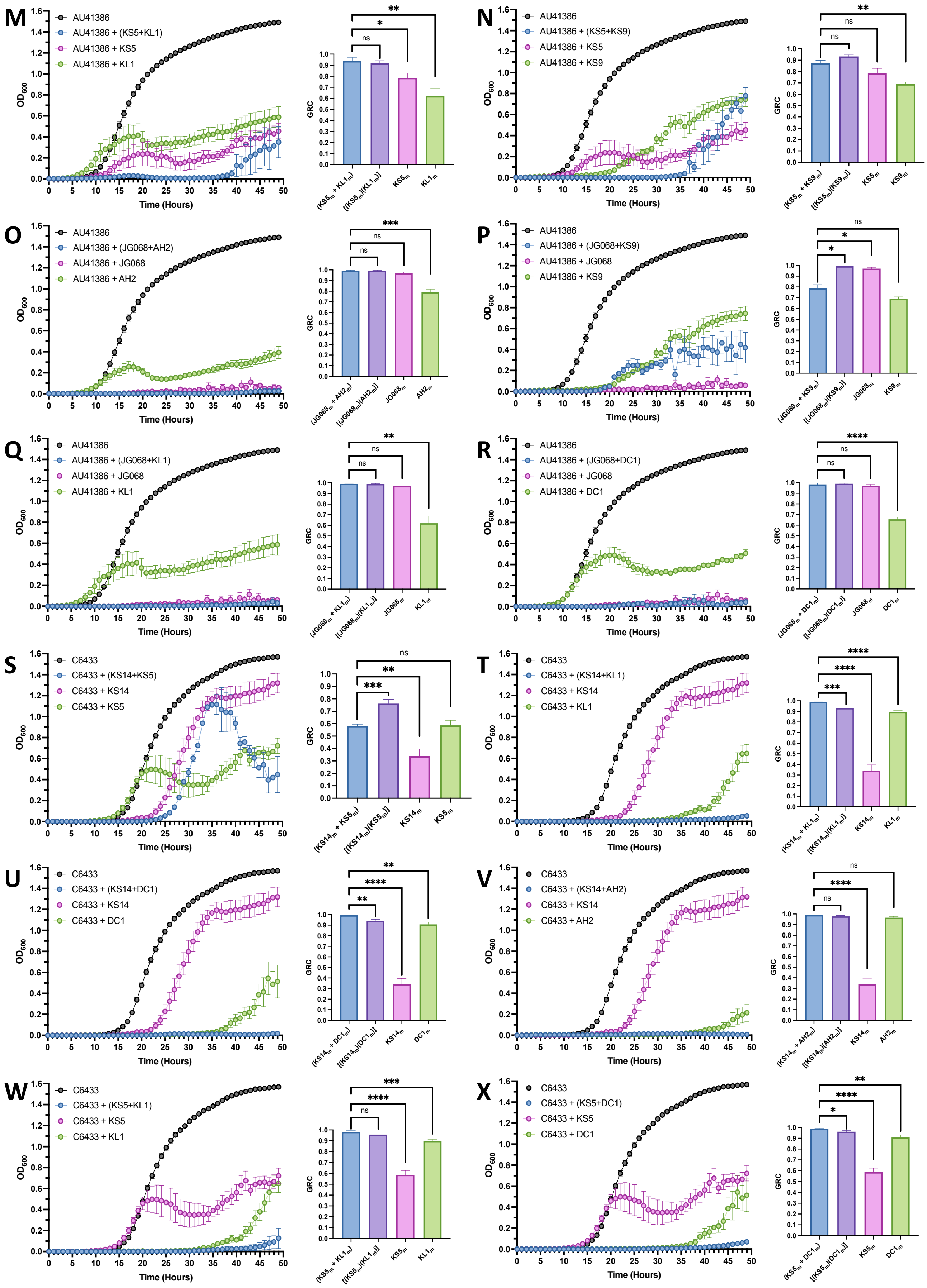
M
AU41386
AU41386 + (KS5+KL1)
AU41386 + KS5
AU41386 + KL1
N
AU41386
AU41386 + (KS5+KS9)
AU41386 + KS5
AU41386 + KS9
O
AU41386
AU41386 + (JG068+AH2)
AU41386 + JG068
AU41386 + AH2
P
AU41386
AU41386 + (JG068+KS9)
AU41386 + JG068
AU41386 + KS9
Q
AU41386
AU41386 + (JG068+KL1)
AU41386 + JG068
AU41386 + KL1
R
AU41386
AU41386 + (JG068+DC1)
AU41386 + JG068
AU41386 + DC1
S
C6433
C6433 + (KS14+KS5)
C6433 + KS14
C6433 + KS5
T
C6433
C6433 + (KS14+KL1)
C6433 + KS14
C6433 + KL1
U
C6433
C6433 + (KS14+DC1)
C6433 + KS14
C6433 + DC1
V
C6433
C6433 + (KS14+AH2)
C6433 + KS14
C6433 + AH2
W
C6433
C6433 + (KS5+KL1)
C6433 + KS5
C6433 + KL1
X
C6433
C6433 + (KS5+DC1)
C6433 + KS5
C6433 + DC1
OD600
Time (Hours)
GRC

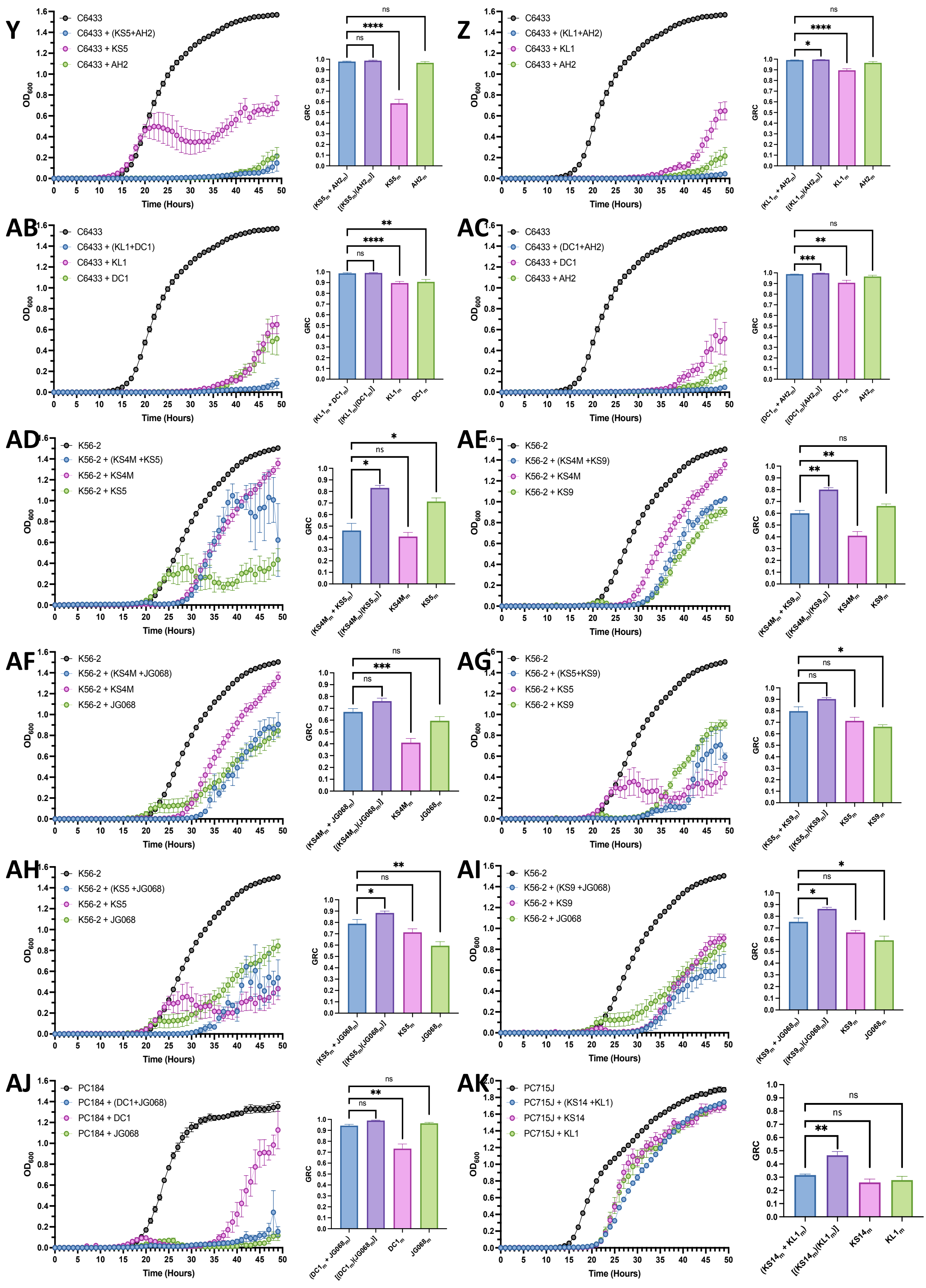
Y
C6433
C6433 + (KS5+AH2)
C6433 + KS5
C6433 + AH2
Z
C6433
C6433 + (KL1+AH2)
C6433 + KL1
C6433 + AH2
AB
C6433
C6433 + (KL1+DC1)
C6433 + KL1
C6433 + DC1
AC
C6433
C6433 + (DC1+AH2)
C6433 + DC1
C6433 + AH2
AD
K56-2
K56-2 + (KS4M +KS5)
K56-2 + KS4M
K56-2 + KS5
AE
K56-2
K56-2 + (KS4M +KS9)
K56-2 + KS4M
K56-2 + KS9
AF
K56-2
K56-2 + (KS4M +JG068)
K56-2 + KS4M
K56-2 + JG068
AG
K56-2
K56-2 + (KS5+KS9)
K56-2 + KS5
K56-2 + KS9
AH
K56-2
K56-2 + (KS5 +JG068)
K56-2 + KS5
K56-2 + JG068
AI
K56-2
K56-2 + (KS9 +JG068)
K56-2 + KS9
K56-2 + JG068
AJ
PC184
PC184 + (DC1+JG068)
PC184 + DC1
PC184 + JG068
AK
PC715J
PC715J + (KS14 +KL1)
PC715J + KS14
PC715J + KL1
OD600
Time (Hours)
GRC

Supplementary Figure 6: Interactions between Bcc-targeting Phages. Bacterial growth curves (left) and treatment GRC bar charts (right) for two-phage combinations on *Burkholderia cenocepacia* strains **AU41264** [KS14+KL1 (**A**), KS14+DC1 (**B**), KL1+DC1 (**C**)], **AU41386** [AH2+KL1 (**D**), AH2+DC1 (**E**), AH2+KS9 (**F**), KL1+DC1 (**G**), KL1+KS9 (**H**), DC1+KS9 (**I**), KS5+AH2 (**J**), KS5+DC1 (**K**), KS5+JG068 (**L**), KS5+KL1 (**M**), KS5+KS9 (**N**), JG068+AH2 (**O**), JG068+KS9 (**P**), JG068+KL1 (**Q**), JG068+DC1 (**R**)], **C6433** [(KS14+KS5 (**S**), KS14+KL1 (**T**), KS14+DC1 (**U**), KS14+AH2 (**V**), KS5+KL1 (**W**), KS5+DC1 (**X**), KS5+AH2 (**Y**), KL1+AH2 (**Z**), KL1+DC1 (**AB**), DC1+AH2 (**AC**)], **K56-2** [(KS4M+KS5 (**AD**), KS4M+KS9 (**AE**), KS4M+JG068 (**AF**), KS5+KS9 (**AG**), KS5+JG068 (**AH**), KS9+JG068 (**AI**)], **PC184** [DC1+JG068 (**AJ**)], and **PC715J** [KS14+KL1 (**AK**)]. Purple bars represent the product of the individual effectivenesses of the component phages, as computed using the right-hand side of **equation 11**. Black line represents bacterial growth without phage. Blue lines and bars represent bacterial growth with and GRC of the phage pair, while pink and green represent bacterial growth with and GRC of the individual phages. Bars represent standard error of the mean of at least three biological replicates. Statistically significant differences between groups were assessed using Student's T-tests (with Welch's correction for unequal variances), and **** indicates $p < 0.0001$, *** indicates $p < 0.001$, ** indicates $p < 0.01$, * indicates $p < 0.05$, while ns indicates $p > 0.05$.

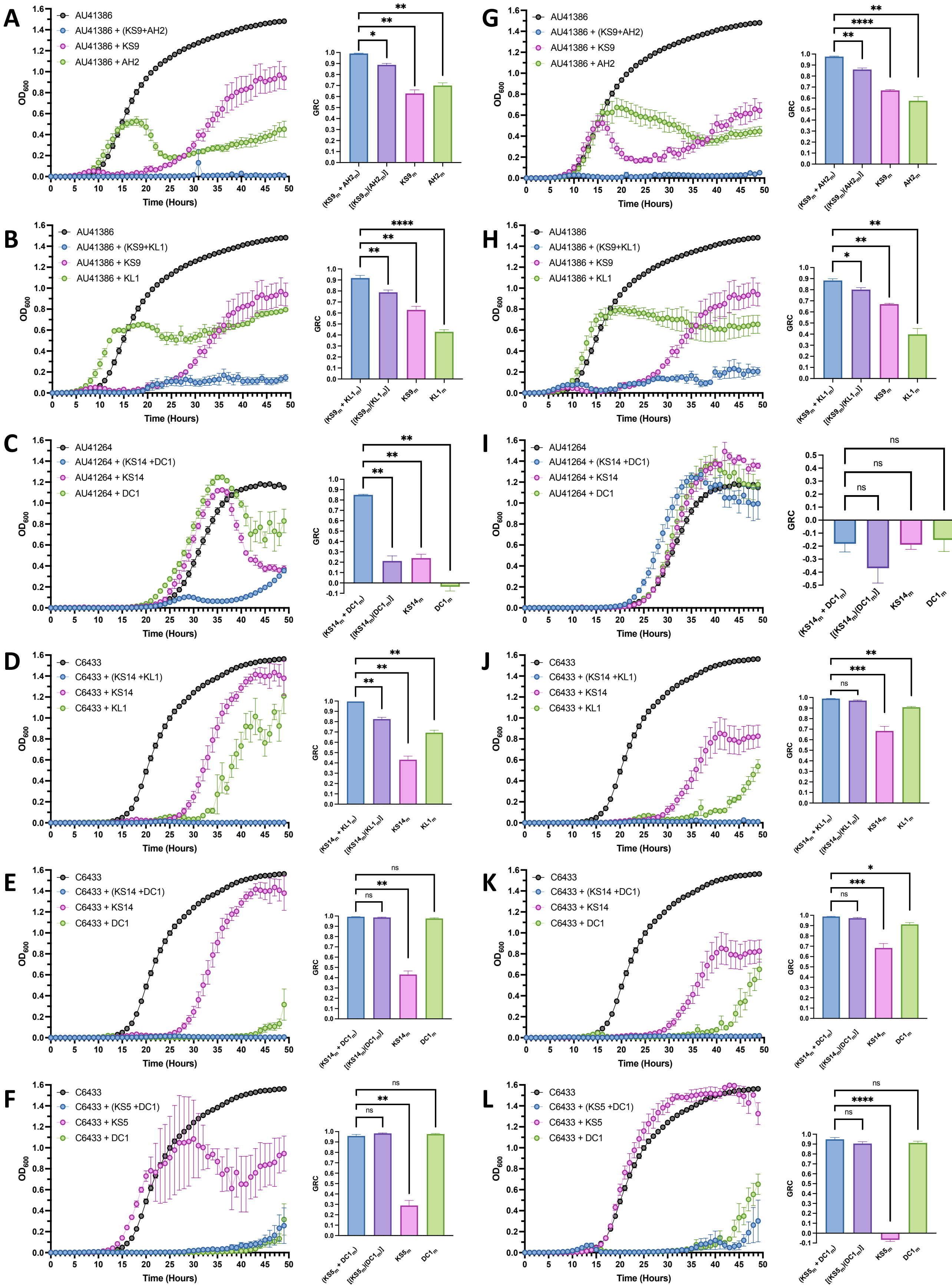
A
AU41386
AU41386 + (KS9+AH2)
AU41386 + KS9
AU41386 + AH2
OD600
Time (Hours)
GRC
(KS9m + AH2m)
[(KS9m)(AH2m)]
KS9m
AH2m
*
**
**
B
AU41386
AU41386 + (KS9+KL1)
AU41386 + KS9
AU41386 + KL1
(KS9m + KL1m)
[(KS9m)(KL1m)]
KL1m
**
**
****
C
AU41264
AU41264 + (KS14 +DC1)
AU41264 + KS14
AU41264 + DC1
(KS14m + DC1m)
[(KS14m)(DC1m)]
KS14m
DC1m
**
**
**
D
C6433
C6433 + (KS14 +KL1)
C6433 + KS14
C6433 + KL1
(KS14m + KL1m)
[(KS14m)(KL1m)]
**
**
**
E
C6433
C6433 + (KS14 +DC1)
C6433 + KS14
C6433 + DC1
ns
**
ns
F
C6433
C6433 + (KS5 +DC1)
C6433 + KS5
C6433 + DC1
(KS5m + DC1m)
[(KS5m)(DC1m)]
KS5m
ns
**
ns
G
**
**
****
H
*
**
**
I
ns
ns
ns
J
ns
***
**
K
ns
***
*
L
ns
****
ns

Supplementary Figure 7: Interactions between Bcc-targeting Phages at lower MOIs. Bacterial growth curves (left) and treatment GRC bar charts (right) for the following phage combinations at MOIs 100 and 1, respectively: AH2 & KS9 (**A**, **G**) and KL1 & KS9 (**B**, **H**) on *Burkholderia cenocepacia* AU41386; KS14 & DC1 (**C**, **I**) on *Burkholderia cenocepacia* AU41264; KS14 & KL1 (**D**, **J**), KS14 & DC1 (**E**, **K**) and KS5 & DC1 (**F**, **L**) on *Burkholderia cenocepacia* C6433. Purple bars represent the product of the individual effectivenesses of the component phages, as computed using the right-hand side of **equation 11**. The black line represents bacterial growth without phage. Blue lines and bars represent bacterial growth with and GRC of the phage pair, while pink and green represent bacterial growth with and GRC of the individual phages. Bars represent standard error of the mean of at least three biological replicates. Statistically significant differences between groups were assessed using Student's T-tests (with Welch's correction for unequal variances), and **** indicates $p < 0.0001$, *** indicates $p < 0.001$, ** indicates $p < 0.01$, * indicates $p < 0.05$, while ns indicates $p > 0.05$.

SUPPLEMENTARY TABLE 1: *Burkholderia cepacia* complex (Bcc) species & strains used in this study

Species & strain	Source [a]	LD_{50} in *G. mellonella*	Bacterial Inoculum	Phage Inoculum	# phage hits	# phages over EPA threshold [b]	# phages over GRC threshold [c]	# pairwise combinations	Reference or source
B. cenocepacia									
C6433	CF, Canada	3.0 x 10^4 CFU	10^5 CFU	10^4 – 10^9 PFU	6	6	5	10	(41)
K56-2	CF-e, Canada	9.0 x 10^2 CFU	10^3 CFU	10^2 – 10^7 PFU	7	7	4	6	(41)
PC715J	CF, Canada	4.0 x 10^3 CFU	10^5 CFU	10^4 – 10^9 PFU	4	3	2	1	(41)
AU41264	CF, USA	No data	10^5 CFU	10^4 – 10^9 PFU	3	3	3	3	this study
AU41386	CF, USA	No data	10^5 CFU	10^4 – 10^9 PFU	6	6	6	15	this study
PC184	CF-e, USA	No data	10^5 CFU	10^4 – 10^9 PFU	2	2	2	1	(78)
J2315	CF-e, UK	1.0 x 10^5 CFU	10^5 CFU	10^4 – 10^9 PFU	1	1	1	0	(41)
K63-3	CF, Canada	No data	10^5 CFU	10^4 – 10^9 PFU	2	1	1	0	(79)
CEP511	CF, Australia	8.0 x 10^4 CFU	10^5 CFU	10^4 – 10^9 PFU	1	1	1	0	(41)
B. ambifaria									
LMG 19467	CF, Australia	8.0 x 10^5 CFU	10^5 CFU	10^4 – 10^9 PFU	3	2	0	0	(41)
LMG 17828	Corn Roots	No data	10^5 CFU	10^4 – 10^9 PFU	1	1	1	0	(80)
B. multivorans									
C5393	CF, Canada	3.0 x 10^6 CFU	10^5 CFU	10^4 – 10^9 PFU	1	1	1	0	(41)
C5274	CF, Canada	1.0 x 10^6 CFU	10^5 CFU	10^4 – 10^9 PFU	1	0	0	0	(41)
B. stabilis									
LMG 14294	CF, Belgium	2.0 x 10^6 CFU	10^5 CFU	10^4 – 10^9 PFU	1	1	1	0	(41)
B. dolosa									
LMG21443	Rhizosphere	4.0 x 10^4 CFU	10^5 CFU	10^4 – 10^9 PFU	1	0	0	0	(41)
AU0158	CF, USA	No data	10^5 CFU	10^4 – 10^9 PFU	1	1	1	0	(81)
CEP021	CF, USA	No data	10^5 CFU	10^4 – 10^9 PFU	1	1	1	0	(80)
**Total:**					**42**	**37**	**30**	**36**	

[a] Abbreviations: CF, cystic fibrosis infection; CF-e, strain that has spread epidemically among CF patients.

[b] In this study, the EPA threshold is arbitrarily set to EPA $\geq$ -5

[c] In this study, the GRC threshold is arbitrarily set to GRC $\geq$ 0.05

SUPPLEMENTARY TABLE 2: *Burkholderia cepacia* complex (Bcc) – targeting Bacteriophages used in this study

Bacteriophage	Morphology	Source	Receptor	Lifestyle	Host Range **[#Hosts]**[a]	Reference
AH2	*Siphoviridae*	*Nandina* sp. soil	Unknown, not LPS	Lysogeny-capable; integrates	*B. cenocepacia* (C6433, K56-2, PC715J, AU41386, K63-3) **[5 hosts]**	(8, 43)
DC1	*Podoviridae*	*Dracaena* sp. soil	Unknown	Lysogeny-capable; integrates	*B. cenocepacia* (C6433, K56-2, AU41264, AU41386, PC184, CEP511) **[6 hosts]**	(8, 44)
JG068	*Podoviridae*	Sewage	LPS O-antigen	Obligately lytic	*B. cenocepacia* (K56-2, AU41386, PC184), *B. stabilis* (LMG 14294), *B. dolosa* (AU0158, CEP021). **[6 hosts]**	(8, 36)
KL1	*Siphoviridae*	Sewage	Unknown, not LPS	Lysogeny-capable	*B. cenocepacia* (C6433, K56-2, PC715J, AU41264, AU41386, K63-3) **[6 hosts]**	(8, 43)
KS4M	*Myoviridae*	Mutant of DK4/BcepMu/KS4	Unknown	Lysogeny-capable	*B. cenocepacia* (C6433, K56-2) *B. ambifaria* (LMG 19467) **[3 hosts]**	(8, 42)
KS5	*Myoviridae*	Onion rhizosphere	LPS (lipid A proximal element)	Lysogeny-capable; integrates at 3' end of AMP nucleosidase	*B. cenocepacia* (C6433, K56-2, PC715J, AU41386, J2315), *B. ambifaria* (LMG 19467) **[6 hosts]**	(8, 42)
KS9	*Siphoviridae*	Lysogen of *B. pyrrocinia* LMG 21824	LPS (lipid A distal element)	Lysogeny-capable; integrates into 3' end of GTP cyclohydrase II gene	*B. cenocepacia* (K56-2, AU41386), *B. ambifaria* (LMG 19467) **[3 hosts]**	(8, 42)
KS14	*Myoviridae*	*Dracaena sp*. soil	Unknown	Lysogeny-capable; forms phagemid	*B. cenocepacia* (C6433, PC715J, AU41264), *B. ambifaria* (LMG 17828), *B. multivorans* (C5393, C5274*),* *B. dolosa* (LMG 21443) **[7 hosts]**	(8, 12)

[a] These are the host ranges reported *in this study*, and differ from the host ranges reported when the phages were first isolated, which are listed comprehensively in (8). A bacterial strain was considered a viable host for a given phage if the phage-host pair yielded an EPA value ≥ -7.

SUPPLEMENTARY TABLE 3: Goodness-of-Fit and Significance Values for Figure 5A and Supplementary Figures 4 & 5

Figure	Analysis	Goodness of Fit	Significance [a]
**Figure 5A:** Correlation between $\boldsymbol{GRC_{x_m}}$ and $f_{(lys)}$ at the maximum available MOI	Linear regression (all data points)	$R^2 = 0.03$	$p > 0.05$ (ns)
	Linear regression (above GRC threshold)	$R^2 = 0.27$	$p = 0.0033$ (**)
	Linear regression (above GRC threshold, excluding outlier group)	$R^2 = 0.67$	$p < 0.0001$ (****)
	Linear regression (below GRC threshold)	$R^2 = 0.28$	$p > 0.05$ (ns)
**Supplementary Figure 4A:** Correlation between EPA and $\boldsymbol{GRC_x}$	Linear regression (all data points)	$R^2 = 0.18$	$p = 0.0083$ (**)
**Supplementary Figure 4B:** Correlation between EPA and $\boldsymbol{GRC_{x_{max}}}$	Linear regression (all data points)	$R^2 = 0.14$	$p = 0.0211$ (*)
**Supplementary Figure 5:** Lack of Significant Correlation between $\boldsymbol{GRC_x}$ and $\boldsymbol{f_{(lys)}}[\boldsymbol{x}]_{(max)}$	Linear regression (all data points)	$R^2 = 0.001$	$p > 0.05$ (ns)
	Linear regression (above GRC threshold)	$R^2 = 0.10$	$p > 0.05$ (ns)
	Linear regression (below GRC threshold)	$R^2 = 0.07$	$p > 0.05$ (ns)

[a] Significance refers to whether or not the slope of the line-of-best-fit is significantly different from zero.

Supplementary Data References

78. Mahenthiralingam E, Coenye T, Chung JW, Speert DP, Govan JRW, Taylor P, Vandamme P. 2000. Diagnostically and experimentally useful panel of strains from the Burkholderia cepacia complex. J Clin Microbiol 38: 910–913.https://doi.org/10.1128/JCM.38.2.910-913.2000.

79. Darling P, Chan M, Cox AD, Sokol PA. 1998. Siderophore production by cystic fibrosis isolates of Burkholderia cepacia. Infect Immun 66:874–877. https://doi.org/10.1128/IAI.66.2.874-877.1998.

80. Cardona ST, Wopperer J, Eberl L, Valvano MA. 2005. Diverse pathogenicity of Burkholderia cepacia complex strains in the Caenorhabditis elegans host model. FEMS Microbiol Lett 250:97–104. https://doi.org/10.1016/j.femsle.2005.06.050.

81. Winsor G, Khaira B, Van Rossum T, Lo R, Whiteside M, Brinkman F. 2008. The Burkholderia Genome Database: facilitating flexible queries and comparative analyses. Bioinformatics 24:2803–2804. https://doi.org/10.1093/bioinformatics/btn524.
